# What Motivates Consumer Food Safety Perceptions and Beliefs? A Scoping Review in BRICS Countries

**DOI:** 10.3390/foods11030432

**Published:** 2022-02-01

**Authors:** Luis D’Avoglio Zanetta, Raísa Moreira Dardaque Mucinhato, Mariana Piton Hakim, Elke Stedefeldt, Diogo Thimoteo da Cunha

**Affiliations:** 1 Multidisciplinary Food and Health Laboratory, School Applied Sciences, State University of Campinas (UNICAMP), Limeira 13484-350, Brazil; luis.dzanetta@hotmail.com (L.D.Z.); pitonmariana@gmail.com (M.P.H.); 2 Postgraduate Program in Nutrition, Universidade Federal de São Paulo (UNIFESP), São Paulo 11015-020, Brazil; raisadardaque@gmail.com; 3 Department of Preventive Medicine, Universidade Federal de São Paulo (UNIFESP), São Paulo 11015-020, Brazil; elke.stedefeldt@unifesp.br

**Keywords:** foodborne disease, consumer, risk perception, Brazil, China, Russia, South Africa, India

## Abstract

This scoping review aimed to map the main evidence in the existing literature regarding consumer perceptions and beliefs regarding food safety in the context of BRICS (Brazil, Russia, India, China, and South Africa) countries. Articles were searched in the Web of Science, Scopus, and SciELO databases. The last search was performed on November 2021. Only the studies conducted within BRICS countries were included. The synthesis aimed to group similarities in consumer beliefs and perceptions of food safety. After screening, 74 eligible articles were included in the study. Of the 74 studies analyzed, 49 (66.2%) were carried out in China, 14 (18.9%) in Brazil, 5 (6.8%) in India,4 (5.4%) in South Africa, and 2 (2.7%) in Russia. Thirty-three motivators of perceptions and beliefs regarding food safety were identified. Food safety motivators were grouped into three categories: (1) sociodemographic characteristics, (2) cognitive aspects, and (3) other. In the “sociodemographic characteristics” category, the motivator with the highest number of corresponding results was education level (results = 22), followed by income (results = 22), both positive drivers for food safety perceptions. The “cognitive aspects” category comprised the majority of the identified motivators. Concern for food safety (results = 32) and risk perception (results = 30) were the motivators with the highest number of results among all categories and motivators. Finally, the main motivator in the “other” category was place of consumption/purchase (results = 8), focusing on consumers underestimating the risk of having a foodborne disease when eating away from home. China and Brazil are leading the way in studies on this topic. Consumers’ perceptions are influenced by socioeconomic characteristics (e.g., age, gender, education level, income), cognitive aspects (e.g., knowledge, risk perception, food concerns, previous experience with food safety incidents) and other situational factors (e.g., price, place of purchase, traceability).

## 1. Introduction

“Safer food saves lives” is the sentence that introduces a document published by the World Health Organization (WHO) [1] on the global burden of foodborne diseases (FBD). In addition to the FBD burden on the population’s health and economy [2,3,4], consumers want to feel safe while eating. In its different spheres, the government must ensure that the food available for consumption meets safety standards. Fulfilling this responsibility can become increasingly complex, as the world is more interconnected, and food systems change rapidly [5]. Food safety is a central point of discussion of several organizations, health agencies, and political representatives. The WHO has established a reference group called the Foodborne Disease Burden Epidemiology Reference Group. This group estimated that in 2010 alone, 31 food hazards caused 600 million illnesses and 420,000 deaths worldwide [2]. Many countries have established policies to control microbiological, chemical, and physical hazards during the food production stage. However, many of these efforts are invisible to the general population. For example, many consumers cannot adequately measure the risk of FBD when eating at a restaurant [6,7], relying predominantly on external cues (e.g., saloon cleanness, employees’ uniform, brand, etc.) to reach a judgment decision. Consumers rely on sensory characteristics at home to identify whether a food is suitable for consumption [8,9]. Consumers use their own repertoire based on their beliefs, risk perceptions, consequences, and heuristics to make decisions regarding food safety [6,10,11]. Food safety is defined as the concept that food will not harm the consumer when it is prepared or consumed [12]. Thus, the hazard could be known or unknown, salient or latent, and perceived as not dreadful or dreadful to the consumer. For example, Mexicans [13] are distrustful of genetically modified food, but not Brazilians [14].

A recent review elucidated some cognitive aspects (e.g., trust, knowledge, attitudes) and socio-demographic characteristics known to positively influence food safety risk perception [15]. An increase in risk perception reduces consumers’ willingness to buy certain foods, e.g., foods of animal origin and those with a high technological or microbiological risk [15]. However, in this review, the authors note that half of the included studies were from the U.S.A., China, and the UK. The country’s development and characteristics could play a significant role, thus directly and indirectly affecting consumer trust and safety. The role of country characteristics is evident in the systematic review by Liguori et al. (2022) [16], which looked at how food safety concerns affect diets in low- and middle-income countries. The authors concluded that aspects such as ease of access, low prices, and convenience can override consumers’ food safety concerns when choosing meals. In addition, despite their knowledge and concern about food safety, many consumers eat out because of a lack of choice and not necessarily because of a preference for street food.

Some developed countries have recognized health surveillance systems, such as the U.S. (U.S. Food and Drug Administration (FDA) policies) and European countries (European Food Safety Authority (EFSA) policies). Health agencies with health surveillance systems grounded in solid and science-based approaches use the principles of risk analysis to assess, manage, and communicate risks properly [17,18]. However, even developed countries suffer from the underreporting of FBD cases and incidents [19,20]. Countries such as Brazil and India and African countries suffer from greater underreporting and an unfinished food safety agenda [21,22,23]. Still, there is evidence that consumers from Brazil, for example, distrust the institutions responsible for food safety, such as government and health surveillance programs [11]. To date, however, there has been no empirical study or research that has shed light on these factors with a focus on middle-income countries or emerging economies such as the BRICS countries. A few studies on food security initiatives have been conducted, discussing food trade and strategies to fight hunger in BRICS [24,25,26]. Food safety is an integral part of food security [27]. Therefore, it is important to understand the current mechanism behind what influences consumer perceptions in this context. BRICS is an acronym for defining a semi-institutionalized political group of countries: Brazil, Russia, India, China, and South Africa. These countries are large developing countries with substantial economic growth potential [28]. According to World Bank data, BRICS countries are responsible for 24% of the global gross domestic production and 16% of the share in world trade [29], displaying an impressive growth in the early 2000s. Together, these countries represented approximately 40% of the world population in 2021 [30]. Despite having some converging economic characteristics, their culture and interests are divergent [31]. For example, Brazil and Russia are more prominent commodity exporters, whereas China is a large commodity importer [28]. In addition, these countries have very different views regarding politics, nuclear power, energy use, etc. [28]. In contrast, BRICS share some common negative characteristics such as corruption, poverty, economic inequalities, and high illiteracy rates [32]. Social inequalities are critical drivers of FBD. Although we do not have specific FBD estimates for each country, the FBD burden is higher in Southern Africa, South America, Southeast Asia, and the Western Pacific Regions than in North America and Europe [2]. As Nordhagen (2022) [33] pointed out in a review of food safety perspectives and practices, foodborne diseases are becoming increasingly problematic as countries develop and urbanize. Therefore, new food safety studies should focus more on understanding individuals’ motivations, beliefs, and values about food safety in specific cultural contexts.

We hypothesize that different socioeconomic characteristics and cognitive aspects shape food safety perceptions and beliefs within BRICS. To verify this hypothesis, we conducted a scoping review. According to Arksey and O’Malley [34], scoping reviews aim to map critical concepts underpinning a research area and the primary available sources and types of evidence. Considering scientific development, comparing BRICS countries is relevant because of the great potential for collaboration for research [35], including food safety research, as is in line with the United Nations Millennium Development Goals [36] and the development of a global strategy for food safety [5]. A review in this context can help these partnerships and direct efforts toward new research on consumers’ perceptions of food safety. This study aims to map the main evidence in the existing literature regarding the motivators of consumers’ perceptions and beliefs regarding food safety in the context of BRICS countries.

## 2. Materials and Methods

This review was organized following the preferred reporting items for systematic reviews and meta-analyses [PRISMA] extension for scoping reviews (PRISMA-ScR) [37]. The PRISMA-ScR checklist was used to check all 20 essential reporting items [37]. PRISMA is a guide that aims to make a review transparent. Reviews that follow PRISMA enable readers to more easily assess the appropriateness of the methods and the trustworthiness of the results [38].

### 2.1. Research Question

This review was guided by the question, “What are the main motivators of food safety perceptions and beliefs of consumers in BRICS countries?” In this study, a scoping review was designed to map the literature on a particular topic or research area to provide key concepts from the last 20 years [39]. Food safety, in this review, was understood as conditions and practices that preserve the quality of food, that is, the idea that food will not cause harm to the consumer when it is prepared or consumed [12]. Belief was understood as acceptance that a statement is true or an attitude that assumes truth in a specific idea. A motivator was understood as a reason to act or behave in a particular way. In addition, motivators were understood to behave in a bi-directional manner, improving or reducing food safety perception, beliefs, and concerns.

### 2.2. Data Sources and Search Strategy

The initial search was performed on 1 November 2021, in three electronic databases: Web of Science™, Scopus^®^, and SciELO databases. These databases were selected as they are comprehensive and cover a broad range of journals in the food science field. Searches were limited to title, abstract, and keywords, using the following search strategy: ((consum* OR custom*) AND (“food safety”) AND (“risk perception” OR belie* OR percept*) AND (Brazil OR Russia OR India OR China OR “South Africa”) NOT (“food handler”)). Filters were used to restrict the results to papers included only in peer-reviewed journals. No language limits were included in this study. Only articles published between 2003 and 2022 were included in the study. The following inclusion criteria were adopted: (a) adults (≥18 years old), (b) quantitative and qualitative methodological approaches, and (c) face-to-face and non-face-to-face methodological approaches. The following articles were excluded: research that (a) has no full-text available; or (b) was part of “gray” literature (i.e., literature that has not been peer-reviewed).

Studies with multiple countries including one or more BRICS countries were eligible. In this case, only the results involving the BRICS countries were considered. The list of selected studies can be found in Table S1.

### 2.3. Selection of the Analysis Corpus

The identification and selection of relevant articles were carried out by two independent judges (LDZ and RMDM), who are researchers in the field. In case of disagreement, a third judge, an expert in the area (MPH), performed an independent review to determine article relevance. The two independent judges initially analyzed the titles, abstracts, and keywords of all articles to select articles for the eligibility study. 

### 2.4. Eligibility Study and Data Extraction

Articles that progressed to the eligibility study were analyzed entirely by three judges (LDZ, RMDM, and MPH) and then included in the corpus of analysis. The judges who participated in the article selection stage worked independently and extracted relevant data from the articles included in the *corpus of analysis*. A fourth judge was called (DTC) in the case of disagreement between the judges. An experienced researcher (DTC) reviewed all data extractions to ensure data accuracy. The following information was extracted from the articles: country of data collection, year of publication, food safety motivators (e.g., risk perception, knowledge, socioeconomic status, etc.), food hazard, and key results.

Motivators were classified according to their nature: quantitative or qualitative. Quantitative measures were derived from scales, sums, scores, percentages, and counts. Results derived from speeches from interviews and focus groups, written responses, and observations (which do not generate scores) were considered qualitative.

### 2.5. Methodological Quality Appraisal

The methodological quality or risk of bias of the included articles was not assessed. This decision is consistent with the guidance of scoping reviews [37].

### 2.6. Synthesis

The synthesis sought to group similarities in consumers’ beliefs and perceptions of food safety. We grouped the studies by country and summarized the motivators in an Excel sheet. The purpose of the synthesis was to group the motivators of food safety perceptions and behaviors. Each motivator was divided into three major categories: (a) sociodemographic characteristics, (b) cognitive aspects, and (c) other factors. The categories were created a posteriori during the full-text reviews. Finally, we discussed all categories for a final consensus. 

## 3. Results

### 3.1. Screening and Eligibility Results

**Figure 1:**
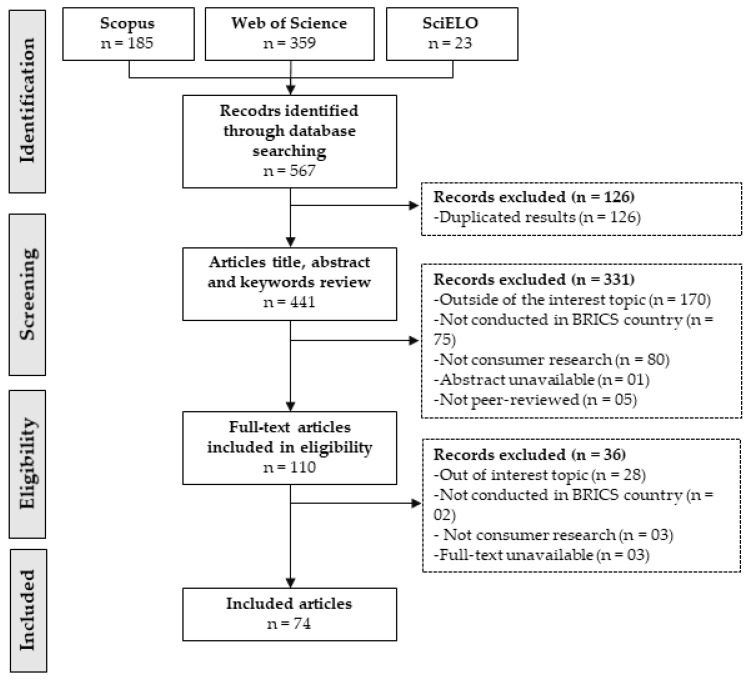
Scoping review flowchart.

Figure 1 shows the number of articles at each stage of the analysis. Initially, 567 studies were identified, 126 of which were duplicates. In the screening stage, 441 had their title, abstract, and keywords analyzed, and 331 articles were excluded, most of which were outside the topic of interest (n = 170). In the eligibility stage, 110 articles were fully read, and 35 were excluded. Finally, the data extraction and results synthesis steps included 74 articles. All included reviews were published between 2003 and 2022, with 89% (n = 67) published after 2012. Of the 74 studies, 83% (n = 62) had a quantitative methodological approach, 13% (n = 9) had a qualitative approach, and 5% (n = 4) had a mixed-approach (qualitative–quantitative). Table S1 presents all selected studies including authors, title, country, and year.

### 3.2. BRICS Differences

**Figure 2:**
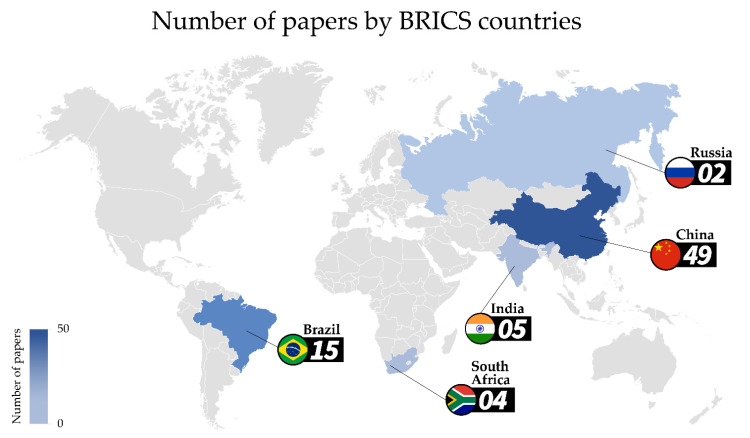
Map chart depicting the number of studies on each BRICS country.

Regarding the prevalence of articles by country, China was predominant (Figure 2). Of the 74 articles analyzed, 49 (65.3%) were carried out in China, 15 (20.0%) in Brazil, 5 (6.7%) in India, 4 (5.3%) in South Africa, and 2 (2.7%) in Russia (Figure 2).

It can be observed that scientific production between countries was heterogeneous. Due to the low number of articles in India, Russia, and South Africa, it was not possible to compare them. However, some research implications and perspectives are discussed.

### 3.3. Hazards and Motivators of Food Safety Perception

Most articles investigated consumers’ perceptions and beliefs regarding FBD, not specifying any specific hazard and instead using generic definitions such as “microbial contamination” (26.2%). Most articles analyzed safety perceptions regarding animal-sourced foods such as milk and dairy (9.8%), pork (6.6%), meat (4.9%), chicken (3.3%), seafood (3.3%), and eggs (1.6%). Some fruits (grapes and apples) and vegetables have also been investigated. Some studies (4.9%) included technological aspects such as food additives, genetically modified organisms (GMOs), and pesticides.

Thirty-three motivators of perceptions and beliefs about food safety were identified with 312 occurrences. In 94% (n = 293) of these occurrences the motivator was assessed quantitatively, and 6% (n = 19) were qualitatively assessed. The category “sociodemographic characteristics” included the following motivators: sex, gender, age, income, education level, race, regionality, and family composition. Meanwhile, the following cognitive aspects were included: knowledge, information, habits, food safety incident experience, media exposure, loss aversion, emotions, concerns regarding food safety, safety perception, risk perception, likelihood and consequences, optimistic bias, self-efficacy, social pressure, subjective norm, protection motivation, response barrier, confidence in the government, confidence in the media, trust in manufacturers’ and retailers’, and trust in certifications. Finally, the “others” category included: price, traceability, place of purchase/consumption, and frequency of consumption. The complete synthesis is provided in the supplementary file (Table S2 for the “sociodemographics” category, Table S3 for the “cognitive factors” category and Table S4 for the “other’ category”). 

Table 1 shows the number of results related to each motivator. Thirty-three motivators of food safety perceptions and beliefs were identified. It should be noted that the same article can present more than one result related to the same motivator (e.g., younger consumers have more risky practices and lower knowledge regarding raw chicken handling [40]).

In the “sociodemographic characteristics” category, the motivator with the highest number of related results was education level (results = 22) and income (results = 22). In general, education level is a positive motivator of food safety. Higher education levels were associated with higher-risk perceptions and beliefs. Some examples elucidate this positive relationship: (a) the greater the education level, the greater the perception of food safety [54] (China); (b) Brazilians with high education levels believe it is safe to consume insects [58] (Brazil); and (c) education level affects food safety awareness of street foods [56] (South Africa). Regarding the income motivator, similar results were observed: (a) High-income consumers are more aware of food safety [9] (South Africa); (b) low-income people are more susceptible to misinformation regarding food safety [46] (China); and (c) the higher the income, the greater the importance of food safety certification in restaurants [55] (Brazil).

Sex (n = 16) was more significant than gender (n = 2) as a motivator of food safety perceptions and beliefs. We used the original definition for sex and gender used in the source reference. However, sex is defined as a biological variation based on physical and physiological characteristics. Gender refers to socially constructed roles based on behaviors, expressions, and identities [110]. In general, sex influences vary according to hazard, food, or associated issues. For example, females have a higher perception of risk concerning GMOs and seafood [25,26,29] (China; Brazil); however, when the consequences of FBD are associated with financial issues, the perception of risk is higher in males [8] (Brazil). The same occurred when reliance on information sources on food safety was considered. Females were more confident than males when this information came from government sources [31] (China), although they were more susceptible to misinformation [27] (China). Females also reported safer practices [24] (South Africa), proven through the predisposition of males to risk practices of de-freezing and handwashing [26] (Brazil). Furthermore, it was observed that gender affects the perception of the food safety of street foods [36] (South Africa); in contrast, gender did not correlate with the sources of risk information used to establish perception of food safety [37] (China).

The age motivator (n = 17) also showed relevance within the “sociodemographic” category. Overall, age influences knowledge, perceptions, and attitudes toward food safety. Searching for information changes with age, and age predicts an enhanced knowledge better than gender and education motivators [40] (South Africa). The younger the age of an individual, the less knowledgeable that individual tends to be regarding food safety [40] (South Africa), and young people, as well as the elderly, are more susceptible to misinformation on this topic [46] (China). It can be observed that with increasing age, the perceptions and practices related to food improve. For example, (a) the older the age, the greater the perception of risk [59] (China); (b) older Brazilians perceive street food to be unclean compared to younger ones [62] (Brazil); (c) younger people assess food safety with greater emphasis on what can be perceived at the time of consumption and not before consumption [43] and (India); (d) the perceived susceptibility to improper food processing is higher in individuals of older ages [48] (China). Regarding attitudes, the younger the age, the more positive the attitude toward certifications related to food safety [55] (Brazil). Riskier practices have also been observed in younger people when handling chicken (e.g., do not handle raw chicken correctly) [40] (South Africa). However, generations X (born between 1966 and 1980), Y (born between 1981 and 1994), and Z (born between 1995 and 2010), in the case of seafood, have a high probability of consuming foods that are unfit for consumption [44] (Brazil).

Other socioeconomic aspects were less frequently identified, such as family composition, regionality, culture and religion, gender, and race.

The cognitive category comprised the majority of the identified motivators. Concern regarding food safety (results = 32) and risk perception (results = 31) were the motivators with the highest number of results among all categories and motivators. Regarding concerns regarding food safety, we can observe, for example, (a) greater concern with food safety and hygiene practices during the COVID-19 pandemic [75] (Brazil); (b) concern regarding pesticides [64] (India); and (c) food safety concerns that are inconsistent with self-reported practices [40] (South Africa). As expected, mixed results were observed for risk perception. Positive results highlighted the following: (a) Consumers’ willingness to pay for safer food had a slight correlation with perceptions of risk [10] (Brazil); and (b) the greater the perception of risk, the greater the search for information on food safety [82,93] (China). Some noteworthy contrary results were also observed: (a) The greater the perception of risk, the greater the intention to choose a restaurant with a high risk of FBD [6] (Brazil); and (b) consumers do not perceive FBD as a significant challenge [9] (South Africa).

Knowledge was shown to be a relevant motivator of perceptions regarding food safety (results = 19); for example, (a) greater knowledge regarding pathogens or professional knowledge regarding food safety increases the risk perception of FBD [49] (China); (b) misinformed individuals disclose erroneous food safety information more often [46] (China); and (c) knowledge regarding food safety positively affects self-protective behavior [70] (China). Knowledge is generally supported by education level or other cognitive aspects, such as risk perception. 

Previous experience with food safety incidents and habits were critical drivers of food safety. The authors state that defensive behavior and risk perception both increase after negative experiences such as an FBD [9,49,61,98], food safety crisis [100], and specific public incidents (e.g., dairy sector in China [53,77]). Negative experiences could increase the consumption of food perceived as safer (e.g., organic food [97]) and reduce the consumption of food perceived as “risky” such as chicken [91]. From the 14 results, 13 were from China, and 1 was from South Africa. Regarding habits, consumers who emphasize hygiene habits, in general, have greater risk perception [44,89] and have better self-protection behavior [70].

Many studies have observed the effect of different types of trust, such as trust in the government, media, manufacturers and retailers, and certification. Positive effects of trust have been observed [9,47,50,77,102]. In contrast, many studies have pointed out that consumers have little or no trust in the government, certifications, and institutions [65,71,77,103]. However, the studies clearly indicate that trust could shape risk perception and willingness to pay for food products. 

Finally, the “others” category comprised 4 of the 34 unidentified motivators. The main motivator of this category was the place of consumption/purchase (results = 8), with emphasis on (a) consumers who underestimate the risk of having an FBD when eating outside the home [10] (Brazil); (b) consumers who prefer to buy meats from a higher-class retail outlet and believe that meats from these places are safer [9] (South Africa); and (c) the lack of structure in the environment (i.e., presence of a refrigerator, running water), which restricts self-protection behaviors [70] (China).

## 4. Discussion

This scoping review made it possible to identify several motivators of food safety perceptions and beliefs. Many articles were selected and analyzed, despite only considering studies from five countries. Several cognitive aspects, as well as socioeconomic and situational characteristics, were identified as significant food safety motivators. The results define the state of the art of consumer surveys in BRICS countries. Research on the socioeconomic and cognitive aspects of food safety is scarce. Many surveys focus on food handlers when discussing professional safety practices [111,112,113], but consumers also play a leading role in food safety.

One of the objectives of this study was to compare the results observed in different BRICS countries, seeking to elucidate convergences and divergences. However, we observed that scientific production in consumer science differs substantially between countries. Despite having the largest population on the planet, China also has solid scientific production. According to SCImago, China was the country with the second highest number of citable documents (CD) (CD = 7,229,532) between 1996 and 2020, only behind the United States [114]. Despite the low number of articles included on the topic of interest in this study, India ranks seventh in terms of citable documents (CD = 1,946,730) [114]. Russia, Brazil, and South Africa are, respectively, 12th (CD = 1,302,809), 14th (CD = 1,067,185), and 35th (CD = 305,649) [114]. When the comparison in SCImago was performed using the “Food Science” category filter, the results differed, showing the potential of China and Brazil, with China 2nd (CD: 70,797), Brazil 5th (CD: 25,947), India 7th (CD: 23,731), South Africa 35th (CD: 4.081), and Russia 43rd (2.463) [114]. 

In addition to the scientific aspect, it is important to highlight how the BRICS countries differ in terms of food policies. The Global Food Security Index (GFSI) measures the variety and nutritional quality of average diets, as well as the safety of food [115]. By sorting the GFSI by the “Quality and Safety” category, as of September 2021, it is possible to observe Brazil in the 13th position (Score: 90.0), Russia in the 23rd (Score: 85.8), South Africa in the 53rd (Score: 72.1), China in the 56th (Score: 71.4), and India in the 74th (Score: 59.1) [116]. This result reflects Brazil’s and Russia’s vocation in food exports [28]. The countries have different vocations and investments, but the importance of the BRICS countries in the global food chain is undeniable. The first suggestion for further research is that researchers from Brazil and China could cooperate with researchers from the BRICS and other developing countries, sharing their expertise in the area of food science.

Of all the results associated with any motivator, 97 emerged from motivators related to socioeconomic characteristics. These results reinforce the importance of socioeconomic factors in different aspects of individuals’ lives. The influence of socioeconomic factors on cognition has been studied in several areas. Quinlan (2013) [117], in his review on FBD incident rates, made it clear that there are patterns of food consumption among a population based on their socioeconomic status, which results in increased exposure to a pathogen. These patterns may be associated with access to more risky foods. It should be noted that although there are studies that seek to identify the role of socioeconomic factors on food safety, data collection from consumers of lower income and education levels is limited [98]. This pattern is due to the advanced language used in research, factors related to filling out questionnaires (e.g., online, social desirability bias), and difficulty in accessing locations. A second suggestion for further research is to investigate the perceptions of consumers of low socioeconomic status. Despite substantial progress in alleviating poverty, BRICS countries still suffer from significant poverty rates, hunger, and FBD [118], aggravated by the pandemic. Studies aimed at investigating populations of a lower socioeconomic status are conducive to developing assertive policies, given their greater vulnerability to FBD and inadequate nutrition.

Knowledge, risk perception, and concerns regarding food safety are the most studied cognitive motivators. The role of knowledge in food safety practices remains controversial [111]. It is widely agreed upon that knowledge alone is not sufficient to change practices [112,119]. As professional food handlers, consumers may have difficulty translating knowledge into practice. Based on low-risk perceptions, consumers search for “shortcuts” when handling food (e.g., performing the procedures as quickly as possible) [111]. This is potentially why we observed many articles dealing with risk perceptions and concerns regarding food safety. Despite this, knowledge is an important driver of food safety as it can motivate positive attitudes [112]. 

Risk perception is the judgment of individuals in characterizing and evaluating a practice or technology identified as risky or dangerous [120]. Risk perception is a cognitive process of decision, generally involving two systems: a) heuristic and intuitive, which occurs without cognitive effort; and b) mental operations that require cognitive effort with either ruled-based, mathematical, or needs-based considerations [121]. The complexity of risk perception can explain these controversial results. Consumers may exert lower risk perception when the risk is perceived as controllable (e.g., FBD, infectious diseases, obesity) [122]. For example, for a foodservice business located in an adequate environment with a positive organizational climate, training, and helpful colleagues, food handlers have a low-risk perception of FBD because they feel safe and in a controlled environment [123]. Therefore, consumers’ perceptions of risk will vary based on any assessed hazards, their knowledge of the subject, and past experiences. A recent review identified that trust, knowledge, subjective characteristics, and socioeconomic status are key drivers of food safety risk perception [15].

Trust in the supply chain has already been stated as an essential driver of food safety risk perception [15]. This result reinforces the importance of the risk analysis. Governments, health surveillance agencies, and stakeholders must be efficient in evaluating, managing, and communicating with consumers regarding food hazards. In 2006, the WHO and Food and Agriculture Organization of the United Nations (FAO) published a guide for food safety authorities regarding risk analysis [124]. This guide provides reliable and valuable strategies for risk communication that can be used in BRICS countries. Effective risk communication can improve consumers’ autonomy, especially in a scenario where the public is increasingly concerned about food risks [125]. Frewer (2009) stated that stakeholders should not only provide information regarding technical risk estimates but also risk information that addresses all the concerns of the targeted group [126].

Finally, we observed some other motivators, such as price, traceability, place of purchase, and frequency of consumption. These factors are directly or indirectly related to socioeconomic and cognitive characteristics. For example, price value is an important motivator of food choices; in general, Brazilian consumers prefer non-expensive food [127]. However, they are willing to pay a premium for safe food when eating out [9]. In this way, traceability information is important in restoring consumer confidence [128]. Consumers are willing to pay a premium to ensure that food can be traced. This premium is higher when traceability is associated with other characteristics, such as extra guarantees of food safety [128]. Consumers interpret the place of purchase/consumption in two main ways: the first is regarding consumers’ characteristics in a given location. For example, higher food safety standards have been attributed to establishments frequented by consumers from higher social classes [9]. The second approach concerns consumers’ cognitive characteristics of risk/situation control. For example, consumers attributed greater safety to the restaurants they chose [10]. This shows that the place of purchase/consumption is essential but indirectly driven by socioeconomic and cognitive factors, as mentioned above.

This study had some limitations. First, the absence of a robust analysis corpus for Russia, South Africa, and India did not allow for assertive comparisons across countries. Furthermore, the vast majority of articles were cross-sectional studies. The relationships were measured through simple analyses, such as a comparison of means and correlations. These data show the importance of more robust studies on this subject using multivariate analysis strategies and qualitative–quantitative designs with data triangulation. 

## 5. Conclusions and Future Perspectives

Using observations from 74 studies, we identified the motivators of food safety perceptions and beliefs considering food safety within the BRICS context. Our findings show that China and Brazil are leading in studies on this subject and would be able to support other countries in research aimed at evaluating, managing, and communicating food safety risks. Thirty-three motivators of perceptions and beliefs regarding food safety were identified. Consumer perceptions are motivated by socioeconomic characteristics (e.g., age, sex, education level, income), cognitive aspects (e.g., knowledge, risk perception, concerns regarding food, previous experience with food safety incidents), and other situational factors (e.g., price, place of purchase, traceability). These findings provide an overview of the primary motivators of food safety perceptions and beliefs in BRICS countries.

According to the initial hypothesis of this study, it can be concluded that cognitive characteristics and aspects can influence food safety beliefs in BRICS countries. Among the observed motivators, it is noteworthy that income, education level, food safety concerns, and risk perception are most strongly associated with food safety behavior change. Nevertheless, it is important to emphasize that there are no studies on this topic in Russia, South Africa, or India, and future studies may uncover new motivators for food safety perceptions and beliefs.

Countries are at different stages of development and have distinct vocations, directly influencing their policies and population. Policies to improve access to education, income, and equity could be essential to improving the perception of food safety among the population. This is fundamental in middle-income countries, such as the BRICS, that still suffer from social inequalities. In contrast, cognitive aspects can be shaped by risk analysis policies, with an investment in assertive and easy-to-understand risk communication.

## Figures and Tables

**Table 1:** Number of related results for each motivator.

Category	Motivator	Number of Related Results *	Papers
Sociodemographic characteristics	Income	22	[9,41,42,43,44,45,46,47,48,49,50,51,52,53,54,55,56]
	Education level	22	[9,40,41,43,44,46,47,48,51,52,54,55,56,57,58,59,60,61]
	Age	17	[40,43,44,45,46,47,48,55,59,62,63,64]
	Sex	16	[9,10,40,42,43,44,46,54,59,60,61,65,66]
	Family composition	07	[43,44,45,50,63,67]
	Regionalism	05	[44,46,58,68]
	Culture and Religion	05	[45,69,70,71]
	Gender	02	[45,56]
	Race	01	[56]
Cognitive aspects	Concerns regarding food safety	32	[40,42,47,50,51,61,63,64,72,73,74,75,76,77,78,79,80,81]
	Risk perception	30	[6,9,10,41,42,44,51,65,68,82,83,84,85,86,87,88,89,90,91,92,93]
	Knowledge	19	[44,49,50,58,61,69,70,77,78,79,89,94,95]
	Safety perception	15	[10,47,50,52,58,91,96,90]
	Experience with food safety incident	14	[9,49,53,61,76,77,89,91,97,98,99,100]
	Habits	11	[7,44,49,64,65,70,89,95,101]
	Media exposure	10	[59,60,67,88]
	Government trust	10	[9,43,65,71,91,102]
	Certification trust	10	[7,9,47,50,65,68,77,103]
	Trust in manufacturers’ and retailers’	09	[9,42,50,67,71,74,104,105]
	Information	07	[45,46,64,91,90,106]
	Loss aversion	05	[10,68,76,107]
	Self-efficacy	03	[46,76]
	Subjective norm	03	[97,106,108]
	Emotions	02	[46,91]
	Likelihood and consequences	02	[10]
	Media trust	02	[86,102]
	Social pressure	01	[93]
	Protection motivation	01	[76]
	Response barrier	01	[76]
Other	Place of consumption and purchase of food	08	[9,10,62,70,95]
	Price	05	[47,50,94]
	Traceability	03	[51,89,96]
	Consumption frequency	02	[50,109]

* The same article can present more than one result related to the same motivator.

## Data Availability

The data presented in this study are available in the supplementary file.

## Supplementary Materials

The following supporting information can be downloaded at: https://www.mdpi.com/article/10.3390/foods11030432/s1. Table S1: details of studies included in the review; Table S2: synthesis table of motivators and effects of “socioeconomic” category; Table S3: synthesis table of motivators and effects of “cognitive factors” category; Table S4: synthesis table of motivators and effects of “socioeconomic” category
