# Supplementary material for: What Motivates Consumer Food Safety Perceptions and Beliefs? A Scoping Review in BRICS Countries
Source: Foods. 2022 Feb 1;11(3):432. doi: 10.3390/foods11030432 (PMC8833883; doi:10.3390/foods11030432)
Supplement: Supplementary file 1 [file foods-11-00432-s001.zip › foods-1542645-supplementary.pdf]

Table S1 - Selected studies including authors, title, country, and year.

	Author	Title	Year	Country
[85]	Gupta, Khanna, and Gupta	A study on the street food dimensions and its effects on consumer attitude and behavioural intentions	2018	India
[106]	Wang and Alexander	The factors of consumer confidence recovery after scandals in food supply chain safety	2018	China
[88]	Ma and Zhang	Analysis of Transmission Model of Consumers' Risk Perception of Food Safety based on Case Analysis	2013	China
[86]	Yan et al.	Are social embeddedness associated with food risk perception under media coverage?	2019	China
[40]	Katiyo et al.	Assessment of safety risks associated with handling chicken as based on practices and knowledge of a group of South African consumers	2019	South Africa
[60]	Zhang et al.	Association of Internet Use with Attitudes Toward Food Safety in China: A Cross-Sectional Study	2019	China
[97]	Wen et al.	Avian Influenza-Factors Affecting Consumers' Purchase Intentions toward Poultry Products	2019	China
[81]	da Fonseca and Salay	Beef, chicken and pork consumption and consumer safety and nutritional concerns in the City of Campinas, Brazil	2008	Brazil
[58]	Schardong et al.	Brazilian consumers' perception of edible insects	2019	Brazil
[63]	Auad et al.	Brazilian Food Truck Consumers' Profile, Choices, Preferences, and Food Safety Importance Perception	2019	Brazil
[62]	Durán-Agüero et al.	Characterization of street food consumption according to age, multicentric study	2018	Argentina, Brazil, Chile, Colombia, Costa Rica, Guatemala, Panama, Paraguay, Peru, Portugal e Uruguay
[87]	Yin et al.	Chinese consumer preferences for fresh produce: Interaction between food safety labels and brands	2018	China
[61]	Liu and Niyongira	Chinese consumers food purchasing behaviors and awareness of food safety	2017	China
[72]	Mirosa, Liu, and Bremer	Chinese consumers' perceptions of food safety cues and maximising the effectiveness of food safety communications	2020	China
[94]	Wang et al.	Chinese consumers' preferences for food quality test/measurement indicators and cues of milk powder: A case of Zhengzhou, China	2019	China
[78]	Shan et al.	Cognitive Biases of Consumers' Risk Perception of Foodborne Diseases in China: Examining Anchoring Effect	2019	China
[79]	Dill et al.	Concerns, attitudes, and opinions of meat buyers in Garanhuns, Pernambuco, Brazil	2021	Brazil

[74]	de Barcellos et al.	Consumer attitudes to different pig production systems: a study from mainland China	2013	China
[71]	Popova et al.	Consumer evaluations of food risk management in Russia	2010	Russia
[95]	Sithole, Bekker, and Mukaratirwa	Consumer knowledge and practices to pork safety in two Taenia solium cysticercosis endemic districts in Eastern Cape Province of South Africa	2020	South Africa
[48]	Wang, Huang, Liang, et al.	Consumer knowledge, risk perception and food-handling behaviors – A national survey in China	2021	China
[73]	Kher et al.	Consumer perceptions of risks of chemical and microbiological contaminants associated with food chains: A cross-national study	2013	- France<br>- Ireland<br>- Poland<br>- Brazil<br>- Netherlands
[42]	Behrens et al.	Consumer purchase habits and views on food safety: A Brazilian study	2010	Brazil
[10]	Zanetta et al.	Consumer risk perceptions concerning different consequences of foodborne disease acquired from food consumed away from home: A case study in Brazil	2022	Brazil
[49]	Wang et al.	Consumer's risk perception of foodborne diseases and high-risk food safety practices in domestic kitchens	2019	China
[76]	Zhu et al.	Consumers' Food Safety Risk Communication on Social Media Following the Suan Tang Zi Accident: An Extended Protection Motivation Theory Perspective	2021	China
[89]	Wang et al.	Consumers' perception toward quality and safety of fishery products, Beijing, China	2009	China
[96]	Feng et al.	Consumers' perceptions of quality and safety for grape products: A case study in Zhejiang Province, China	2012	China
[43]	Ali	Consumers' Perspectives on Mandatory versus Voluntary Provisions of Food Safety Regulations in India	2013	India
[9]	Dastile, Francis, and Muchenje	Consumers' Social Representations of Meat Safety in Two Selected Restaurants of Raymond Mhlaba Municipality in the Eastern Cape, South Africa	2017	South Africa
[50]	Zhang et al.	Consumers' perceptions, purchase intention, and willingness to pay a premium price for safe vegetables: A case study of Beijing, China	2018	China
[69]	Kiran et al.	Consumption pattern, consumer attitude and consumer perception on meat quality and safety in Southern India	2018	India
[44]	Baptista, Rodrigues, and Sant'Ana	Consumption, knowledge, and food safety practices of Brazilian seafood consumersConsumption, knowledge, and food safety practices of Brazilian seafood consumers	2020	Brazil
[64]	Kar and Meena	Customer's decisiveness about food safety apprehensions of dairy products: A consumer-centric approach	2019	India
[70]	Wang et al.	Determinants of consumer food safety self-protection behavior-- an analysis using grounded theory	2020	China
[45]	Han and Liu	Does Information Pattern Affect Risk Perception of Food Safety? A National Survey in China	2018	China

[75]	Rodrigues et al.	Effect of the COVID-19 pandemic on food habits and perceptions: A study with Brazilians	2021	Brazil
[77]	Maitiniyazi and Canavari	Exploring Chinese consumers' attitudes toward traceable dairy products: A focus group study	2020	China
[68]	Lai et al.	Factoring Chinese consumers’ risk perceptions into their willingness to pay for pork safety, environmental stewardship, and animal welfare	2018	China
[51]	Wu et al.	Factors Affecting Consumer Willingness to Pay for Certified Traceable Food in Jiangsu Province of China	2021	China
[46]	Wang et al.	Fake news or bad news? Toward an emotion-driven cognitive dissonance model of misinformation diffusion	2020	China
[91]	Zhou et al.	Fear and trust: How risk perceptions of avian influenza affect Chinese consumers’ demand for chicken	2016	China
[52]	Yormirzoev, Teuber, and Li	Food quality vs food patriotism: Russian consumers’ preferences for cheese after the food import ban	2019	Russia
[102]	Hessel et al.	Food safety behavior and handling practices during purchase, preparation, storage and consumption of chicken meat and eggs	2019	Brazil
[47]	Liu et al.	Food safety concerns and consumer preferences for food safety attributes: Evidence from China	2020	China
[7]	Bai et al.	Food safety in restaurants: The consumer perspective	2019	China
[65]	Si, Regnier-Davies, and Scott	Food safety in urban China: Perceptions and coping strategies of residents in Nanjing	2017	China
[100]	Yi et al.	Food Safety Incident, Public Health Concern, and Risk Spillover Heterogeneity: Avian Influenza Shocks as Natural Experiments in China’s Consumer Markets	2019	China
[53]	Dong and Li	Food safety issues in China: a case study of the dairy sector	2015	China
[54]	Wang et al.	Food safety perception and consumption decision: A positive analysis on individuals' consumer in Tianjin City	2003	China
[93]	Yang et al.	Food safety risk information-seeking intention of wechat users in China	2020	China
[82]	Liao, Luo, and Zhu	Food Safety Trust, Risk Perception, and Consumers' Response to Company Trust Repair Actions in Food Recall Crises	2020	China
[59]	Liu and Ma	Food scandals, media exposure, and citizens' safety concerns: A multilevel analysis across Chinese cities	2016	China
[109]	Bai et al.	Hygienic food handling intention. An application of the Theory of Planned Behavior in the Chinese cultural context	2014	China
[108]	Nie et al.	Influence of Loss Aversion and Income Effect on Consumer Food Choice for Food Safety and Quality Labels	2021	China
[6]	de Andrade et al.	Knowledge and risk perceptions of foodborne disease by consumers and food handlers at restaurants with different food safety profiles	2019	Brazil
[80]	Ortega et al.	Modeling heterogeneity in consumer preferences for select food safety attributes in China	2011	China
[83]	Danelon and Salay	Perceived physical risk and risk-reducing strategies in the consumption of raw vegetable salads in restaurants	2012	Brazil

[110]	Xu, Zheng, and Motamed	Perceived risks and safety concerns about fluid milk among Chinese college students	2010	China
[66]	Veeck, Veeck, and Zhao	Perceptions of Food Safety by Urban Consumers in Nanjing, China	2015	China
[107]	Wu et al.	Public risk perception of food additives and food scares. The case in Suzhou, China	2013	China
[99]	Wang, Diao, and Tou	Research on the Influence Mechanism of Rational Consumers' Food Safety Supervision Satisfaction	2019	China
[103]	Sun	Research on the influencing factors of consumer food safety risk perception	2017	China
[92]	Behrens et al.	Social representations of safety in food services	2015	Brazil
[57]	Rajkumar et al.	Socio-demographic influences on various consumer food safety indices: an empirical study of ethnic Goan pork sausage consumers	2021	India
[55]	Uggioni and Salay	Sociodemographic and knowledge influence on attitudes towards food safety certification in restaurants	2014	Brazil
[105]	Chen	The effects of different types of trust on consumer perceptions of food safety: An empirical study of consumers in Beijing Municipality, China	2013	China
[56]	Asiegbu, Lebelo, and Tabit	The food safety knowledge and microbial hazards awareness of consumers of ready-to-eat street-vended food	2016	South Africa
[84]	Zhao, Deng, and Zhou	The impact of reference effects on online purchase intention of agricultural products The moderating role of consumers' food safety consciousness	2017	China
[98]	Liu and Zheng	The Predictors of Consumer Behavior in Relation to Organic Food in the Context of Food Safety Incidents: Advancing Hyper Attention Theory Within an Stimulus-Organism-Response Model	2019	China
[67]	Maitiniyazi and Canavari	Understanding Chinese consumers' safety perceptions of dairy products: a qualitative study	2021	China
[41]	Wang, Ge, and Ma	Urban Chinese Consumers' Willingness to Pay for Pork with Certified Labels: A Discrete Choice Experiment	2018	China
[104]	Moruzzo et al.	Urban Consumer Trust and Food Certifications in China	2020	China
[101]	Wei-qun et al.	Why Do Consumers Switch in Food Safety Crisis? A View of Regulatory Focus Theory	2015	China

Table S2 – Synthesis table of motivators and effects of "socioeconomic" category

Motivator	Observed effect	Hazard	Country	Study title	Year
**Sex**	Females had a greater perception of food safety	GMO	China	Food safety perception and consumption decision: A positive analysis on individuals' consumer in Tianjin City	2003
		None	China	Food scandals, media exposure, and citizens' safety concerns: A multilevel analysis across Chinese cities	2016
	Females trust more government information on food safety	Chemical, Biological, GMO Hazards	China	Perceptions of Food Safety by Urban Consumers in Nanjing, China	2015
	Males rely more on food safety information online	Chemical, Biological, GMO Hazards	China	Perceptions of Food Safety by Urban Consumers in Nanjing, China	2015
	Males underestimates more the risks of food contamination by chemicals and microorganisms	Food contamination	Brazil	Consumer purchase habits and views on food safety: A Brazilian study	2010
	Male sex has increased risk perception for consequences of Foodborne disease associated with financial issues	Foodborne disease	Brazil	Consumer risk perceptions concerning different consequences of Foodborne disease acquired from food consumed away from home: A case study in Brazil	2022
	Women reported following more safe practices	Foodborne disease - Raw chicken	South Africa	Assessment of safety risks associated with handling chicken as based on practices and knowledge of a group of South African consumers	2019
	Men and rural residents had higher assessments regarding food safety	None	China	Association of Internet Use with Attitudes Toward Food Safety in China: A Cross-Sectional Study	2019
	Male consumers have a significant positive relationship with the voluntary mechanism for regulating food safety	Regulation	India	Consumers' Perspectives on Mandatory versus Voluntary Provisions of Food Safety Regulations in India	2013
	Consumers who are responsible for the diet of families are more sensitive to food safety	Foodborne disease in meat	South Africa	Consumers' Social Representations of Meat Safety in Two Selected Restaurants of Raymond Mhlaba Municipality in the Eastern Cape, South Africa	2017
	Male respondents have a higher relative risk when compared to females (they are	Seafood	Brazil	Consumption, knowledge, and food safety practices of Brazilian seafood consumers	2020

	more likely to thaw seafood at room temperature)				
	Male respondents are more likely to wash their hands only with water, believing that the chances of getting sick due to raw seafood consumption are low	Seafood	Brazil	Consumption, knowledge, and food safety practices of Brazilian seafood consumers	2020
	Male respondents are less likely to have heard about Vibrio, Listeria, and Salmonella as bacterial pathogens potentially present in seafood	Seafood	Brazil	Consumption, knowledge, and food safety practices of Brazilian seafood consumers	2020
	Females had a higher perception of risk	Seafood	Brazil	Consumption, knowledge, and food safety practices of Brazilian seafood consumers	2020
	Females are more susceptible to food safety misinformation	Dissemination of misinformation	China	Fake news or bad news? Toward an emotion-driven cognitive dissonance model of misinformation diffusion	2020
	Females are more concerned about shelf life, food color and nutritional content	Foodborne disease - awareness	China	Chinese consumers food purchasing behaviors and awareness of food safety	2017
	Females are better at knowing the processing of leftovers and thawing raw meat	Foodborne disease	China	Consumer knowledge, risk perception and food-handling behaviors – A national survey in China	2021
**Gender**	Gender affects how consumers perceive the safety of food sold on the street	Microbiological Hazard	South Africa	The food safety knowledge and microbial hazards awareness of consumers of ready-to-eat street-vended food	2016
	Gender is not significantly correlated with perception of risk to food safety	Sources of risk information	China	Does Information Pattern Affect Risk Perception of Food Safety? The National Survey in China	2018
**Race**	Race affects how consumers perceive the safety of food sold on the street	Microbiological Hazard	South Africa	The food safety knowledge and microbial hazards awareness of consumers of ready-to-eat street-vended food	2016
**Age**	The older the age, the greater the perception of eating risk	None	China	Food scandals, media exposure, and citizens' safety concerns: A multilevel analysis across Chinese cities	2016
	The lower the age, the lower the positive attitude towards restaurant certification	Restaurant certification	Brazil	Sociodemographic and knowledge influence on attitudes towards food safety certification in restaurants	2014

	The youngest (18–29 years) have riskier practices	Foodborne disease - raw chicken	South Africa	Assessment of safety risks associated with handling chicken as based on practices and knowledge of a group of South African consumers	2019
	The youngest (18-29 years) showed that they had much less knowledge than respondents in other age groups, except those aged 30-39 years	Foodborne disease - raw chicken	South Africa	Assessment of safety risks associated with handling chicken as based on practices and knowledge of a group of South African consumers	2019
	Age predicted better practices and knowledge than gender and level of education	Foodborne disease - raw chicken	South Africa	Assessment of safety risks associated with handling chicken as based on practices and knowledge of a group of South African consumers	2019
	Younger consumers emphasize experience attributes (search attributes = evaluates food safety and quality attributes at the time of consumption).	Regulation	India	Consumers' Perspectives on Mandatory versus Voluntary Provisions of Food Safety Regulations in India	2013
	Age positively correlates with voluntary (factor) mechanism for regulating food safety.	Regulation	India	Consumers' Perspectives on Mandatory versus Voluntary Provisions of Food Safety Regulations in India	2013
	Generations X, Y, and Z are more likely to consume raw or undercooked seafood	Seafood	Brazil	Consumption, knowledge, and food safety practices of Brazilian seafood consumers	2020
	Generations X and Y are more likely to believe that they are unlikely to get older from raw seafood consumption	Seafood	Brazil	Consumption, knowledge, and food safety practices of Brazilian seafood consumers	2020
	Generation Z is more likely to wash hands only with water	Seafood	Brazil	Consumption, knowledge, and food safety practices of Brazilian seafood consumers	2020
	The source of information for knowledge about food items changes according to age. Adults and young people search cookbooks and older people seek information from food experts	Milk and Dairy	India	Customer's decisiveness about food safety apprehensions of dairy products: A consumer-centric approach	2019
	Young urban consumers (with higher education) tend to	Sources of risk information	China	Does Information Pattern Affect Risk Perception of Food Safety? The National Survey in China	2018

	perceive a higher level of risk concerning food safety				
	Younger people (25 years or younger) and the elderly (over 65 years) are more susceptible to misinformation in food safety	Dissemination of misinformation	China	Fake news or bad news? Toward an emotion-driven cognitive dissonance model of misinformation diffusion	2020
	Middle-aged and older people are more concerned about the fuji apple	Fuji Apple	China	Food safety concerns and consumer preferences for food safety attributes: Evidence from China	2020
	Age increases the perception linked to food dirt, and perception linked to partially cleaned foods decreases	Foodborne disease - street food	Brazil	Characterization of street food consumption according to age, multicentric study	2018
	Consumer susceptibility to improper processing of leftovers and thawing raw meat increases with increasing age	Foodborne disease	China	Consumer knowledge, risk perception and food-handling behaviors – A national survey in China	2021
	The younger age group (≤30) has a greater perception of the importance of food safety, and older people (≥50), lower perception	Foodborne disease - Food truck	Brazil	Brazilian Food Truck Consumers' Profile, Choices, Preferences, and Food Safety Importance Perception	2019
**Income**	The higher income, the greater the perception of food safety	GMO	China	Food safety perception and consumption decision: A positive analysis on individuals' consumer in Tianjin City	2003
	The higher the income, the lower the importance of restaurant certification	Restaurant certification	Brazil	Sociodemographic and knowledge influence on attitudes towards food safety certification in restaurants	2014
	Monthly income affects how consumers perceive the safety of food sold on the street	Microbiological Hazard	South Africa	The food safety knowledge and microbial hazards awareness of consumers of ready-to-eat street-vended food	2016
	The higher the income, the higher the provision of payment for certified products	Certified pork	China	Urban Chinese Consumers' Willingness to Pay for Pork with Certified Labels: A Discrete Choice Experiment	2018
	Housewives in the higher income group seem to prefer frozen chicken as a way to ensure food safety	Food contamination	Brazil	Consumer purchase habits and views on food safety: A Brazilian study	2010

	High-income (generally highly educated) groups have a higher perception of foodborne disease risk and are more skilled and faster at researching and mastering food safety knowledge	Foodborne disease	China	Consumer's risk perception of Foodborne diseases and high-risk food safety practices in domestic kitchens	2019
	Consumers with higher monthly income emphasize the shape/size, colorn and variety of food products as an attribute of food quality and safety.	Regulation	India	Consumers' Perspectives on Mandatory versus Voluntary Provisions of Food Safety Regulations in India	2013
	Consumers with higher monthly income and consumers who have wage-earners emphasize credit attributes (credence attributes = evaluation of food quality and safety attributes by regulatory certifications and practices).	Regulation	India	Consumers' Perspectives on Mandatory versus Voluntary Provisions of Food Safety Regulations in India	2013
	High-income consumers are more aware of food safety	Foodborne disease in meats	South Africa	Consumers' Social Representations of Meat Safety in Two Selected Restaurants of Raymond Mhlaba Municipality in the Eastern Cape, South Africa	2017
	Family income had a significant positive influence on the willingness to pay for a premium price, but not on the intention to purchase safe vegetables.	Vegetables	China	Consumers' perceptions, purchase intention, and willingness to pay a premium price for safe vegetables: A case study of Beijing, China	2018
	High-income consumers are more likely to consume raw or undercooked seafood	Seafood	Brazil	Consumption, knowledge, and food safety practices of Brazilian seafood consumers	2020
	Consumer income has a significant positive effect on the frequency of seafood consumption	Seafood	Brazil	Consumption, knowledge, and food safety practices of Brazilian seafood consumers	2020
	Higher middle-income citizens spend more time transporting seafood	Seafood	Brazil	Consumption, knowledge, and food safety practices of Brazilian seafood consumers	2020

	Household income is not significantly correlated with the perception of risk to food safety	Sources of risk information	China	Does Information Pattern Affect Risk Perception of Food Safety? The National Survey in China	2018
	Income has a significant effect on the willingness to pay a maximum amount in traceable foods	Food traceability	China	Factors Affecting Consumer Willingness to Pay for Certified Traceable Food in Jiangsu Province of China	2021
	Low-income people are more susceptible to misinformation in food safety	Dissemination of misinformation	China	Fake news or bad news? Toward an emotion-driven cognitive dissonance model of misinformation diffusion	2020
	With increased income individuals are less likely to prefer internally produced cheeses for quality preference reasons primarily, but also for economic reasons	National x imported cheese	Russia	Food quality vs food patriotism: Russian consumers' preferences for cheese after the food import ban	2018
	People with higher family income are more concerned about fuji apple choices	Apple fuji	China	Food safety concerns and consumer preferences for food safety attributes: Evidence from China	2020
	Reflection on the dairy safety incident of relatively low-income groups is more intense than in high-income groups, and the decrease in consumption of low-income groups is more expected after incidents with milk safety	Dairy	China	Food safety issues in China: a case study of the dairy sector	2015
	Income had significant effects on knowledge, risk perception and normative frequency of raw meat thawing but did not affect the processing of leftovers	Foodborne disease	China	Consumer knowledge, risk perception and food-handling behaviors – A national survey in China	2021
	Average monthly household income between 3001 and 5,000 Yuan attach the greatest importance to the perception of risk of thawing raw meat, while consumers with average monthly household income below 1000 Chinese Yuan are the weakest in the perception	Foodborne disease	China	Consumer knowledge, risk perception and food-handling behaviors – A national survey in China	2021

	of risk of improper handling of raw meat.				
**Education level**	The higher education, the greater the perception of food safety	GMO	China	Food safety perception and consumption decision: A positive analysis on individuals' consumer in Tianjin City	2003
	The higher the schooling, the greater the concern with food safety	None		Food scandals, media exposure, and citizens' safety concerns: A multilevel analysis across Chinese cities	2016
	The higher the education, the higher the KABPI (knowledge, attitudes, beliefs, practices index)	Goan pork sausage	China	Socio-demographic influences on various consumer food safety indices: an empirical study of ethnic Goan pork sausage consumers	2021
	The higher the schooling, the lower the importance of restaurant certification	Restaurant certification	Brazil	Sociodemographic and knowledge influence on attitudes towards food safety certification in restaurants	2014
	Schooling affects how consumers perceive the safety of food sold on the street	Microbiological Hazard	South Africa	The food safety knowledge and microbial hazards awareness of consumers of ready-to-eat street-vended food	2016
	The higher the schooling, the higher the provision of payment for certified products	Certified pork	China	Urban Chinese Consumers' Willingness to Pay for Pork with Certified Labels: A Discrete Choice Experiment	2018
	The higher the schooling, the better informed the factors that affect the safety of chicken meat	Foodborne disease - raw chicken	South Africa	Assessment of safety risks associated with handling chicken as based on practices and knowledge of a group of South African consumers	2019
	The higher the schooling, the greater the concern with food safety		China	Association of Internet Use with Attitudes Toward Food Safety in China: A Cross-Sectional Study	2019
	More educated consumers emphasize credit attributes (credence attributes = evaluation of food quality and safety attributes by regulatory certifications and practices).	Regulation	India	Consumers' Perspectives on Mandatory versus Voluntary Provisions of Food Safety Regulations in India	2013
	High-level educational consumers are more aware of food safety	Foodborne disease in meats	South Africa	Consumers' Social Representations of Meat Safety in Two Selected Restaurants of Raymond Mhlaba Municipality in the Eastern Cape, South Africa	2017
	Graduate consumers are more likely to consume raw or undercooked seafood	Seafood	Brazil	Consumption, knowledge, and food safety practices of Brazilian seafood consumers	2020

	Graduate students are more likely to have heard of Vibrio and Listeria as foodborne pathogens	Seafood	Brazil	Consumption, knowledge, and food safety practices of Brazilian seafood consumers	2020
	Schooling has a significant effect on the willingness to pay a prêmium amount in traceable foods	Food traceability	China	Factors Affecting Consumer Willingness to Pay for Certified Traceable Food in Jiangsu Province of China	2021
	People with less education are more susceptible to misinformation in food safety	Dissemination of misinformation	China	Fake news or bad news? Toward an emotion-driven cognitive dissonance model of misinformation diffusion	2020
	With increased education, individuals are less likely to prefer in-house produced cheeses for quality preference reasons primarily, but also economic reasons	National x imported cheese	Russia	Food quality vs food patriotism: Russian consumers' preferences for cheese after the food import ban	2018
	More educated people are more concerned about the choices of the fuji apple	Apple fuji	China	Food safety concerns and consumer preferences for food safety attributes: Evidence from China	2020
	People with a higher level of education (graduate) believe that insect consumption is safe	Safety of insect consumption	Brazil	Brazilian consumers' perception of edible insects	2019
	lower levels of education affect consumers' knowledge and the type of concern for food safety	Foodborne disease - awareness	China	Chinese consumers food purchasing behaviors and awareness of food safety	2017
	concerns related to product life also differed among respondents with different levels of education	Foodborne disease - awareness	China	Chinese consumers food purchasing behaviors and awareness of food safety	2017
	The higher the level of education, the higher the score of consumers' knowledge about the processing of leftovers and thawing of raw meat.	Foodborne disease	China	Consumer knowledge, risk perception and food-handling behaviors – A national survey in China	2021
	The higher the educational level, the higher the perceived severity concerning the incorrect handling of raw meat; less likely to believe that improper processing of	Foodborne disease	China	Consumer knowledge, risk perception and food-handling behaviors – A national survey in China	2021

	leftovers would lead to adverse consequences;				
	The lower the level of education, the less likely to adopt a normative leftovers processing behavior	Foodborne disease	China	Consumer knowledge, risk perception and food-handling behaviors – A national survey in China	2021
	the level of education does not affect the perceived susceptibility and normative frequency of thawing of raw meat.	Foodborne disease	China	Consumer knowledge, risk perception and food-handling behaviors – A national survey in China	2021
**Family composition**	Consumers who have children or elderly people at home to express more concern about the safety and quality of food in dairy products	dairy products	China	Understanding Chinese consumers' safety perceptions of dairy products: a qualitative study	2021
	Consumers who have more children in the family place more emphasis on experience attributes (search attributes = assesses food safety and quality attributes at the time of consumption).	regulation	India	Consumers' Perspectives on Mandatory versus Voluntary Provisions of Food Safety Regulations in India	2013
	If the family has children under 18, the intention to buy safe vegetables increases	Vegetables	China	Consumers' perceptions, purchase intention, and willingness to pay a premium price for safe vegetables: A case study of Beijing, China	2018
	Families without children are more likely to consume raw or undercooked seafood	Seafood	Brazil	Consumption, knowledge, and food safety practices of Brazilian seafood consumers Consumption, knowledge, and food safety practices of Brazilian seafood consumers	2020
	The presence of children in the family affects the perception of risk of seafood and safety practices	Seafood	Brazil	Consumption, knowledge, and food safety practices of Brazilian seafood consumers Consumption, knowledge, and food safety practices of Brazilian seafood consumers	2020
	Family size is not significantly correlated with perceived food safety risk	Risk information sources	China	Does Information Pattern Affect Risk Perception of Food Safety? The National Survey in China	2018
	childless consumers getting higher perception scores of the importance of food safety	Foodborne disease - Food truck	Brazil	Brazilian Food Truck Consumers' Profile, Choices, Preferences, and Food Safety Importance Perception	2019

Table S3 - Motivators and effects of "cognitive aspects" category

Motivator	Observed effect	Hazard	Country	Study title	Year
**Risk Perception**	The greater the perception of risk, the greater the search for information on food safety risk	None	China	Food safety risk information-seeking intention of wechat users in China	2020
		None	China	Food Safety Trust, Risk Perception, and Consumers' Response to Company Trust Repair Actions in Food Recall Crises	2020
	The greater the perception of risk, the greater the protective behavioral intention	None	China	Food Safety Trust, Risk Perception, and Consumers' Response to Company Trust Repair Actions in Food Recall Crises	2020
	The greater the perception of food safety risk, the less willing to pay	foodborne disease	China	The meta-analytic review of food safety risk perception	2020
	The perception of food safety risk is influenced by subjective characteristics (perceived control, concern, perceived benefit, preference for natural foods) and not by the initial negative attitude	foodborne disease	China	The meta-analytic review of food safety risk perception	2020
	The lower the risk perception, the greater the intention to choose high-risk restaurants for Foodborne disease	foodborne disease	Brazil	Knowledge and risk perceptions of Foodborne disease by consumers and food handlers at restaurants with different food safety profiles	2019
	Consumers with moderate risk perception seek strategies to reduce risk	Salads and raw vegetables at full-service restaurants	Brazil	Perceived physical risk and risk-reducing strategies in the consumption of raw vegetable salads in restaurants	2012
	Greater perception of risk associated with the safety of meat products	food contamination	Brazil	Consumer purchase habits and views on food safety: A Brazilian study	2010
	The high risk perception in relation to the consumption of vegetables and meat seems to justify extreme - and sometimes inappropriate - behaviors applied to improve the level of safety	food contamination	Brazil	Consumer purchase habits and views on food safety: A Brazilian study	2010
	Low risk perception in home environments	food contamination	Brazil	Consumer purchase habits and views on food safety: A Brazilian study	2010
	Risk perception has a mediating role in the relationships in the degree of influence of Reference Groups and online comments in the process of	Purchase of agricultural products	China	The impact of reference effects on online purchase intention of agricultural products The moderating role of consumers' food safety consciousness	2017

	making the purchase decision				
	Low risk perception in restaurants	foodborne disease	Brazil	Consumer risk perceptions concerning different consequences of Foodborne disease acquired from food consumed away from home: A case study in Brazil	2022
	The perception of risk is responsible for the change in attitude towards street food	Street food	India	A study on the street food dimensions and its effects on consumer attitude and behavioral intentions	2018
	Hygienic risk was the most critical perceived risk				
	Consumers can develop a negative perception and perceive the health risk				
	The path (in chain, radiation, chain with radiation and migration) of transmission of risk perception to food safety, leads to the speed of rapid diffusion of risk perception;	food safety risk transmission perception	China	Analysis of Transmission Model of Consumers' Risk Perception of Food Safety based on Case Analysis	2013
	Improved risk perception through increased government credibility, transparency of information and advertising, and improved risk awareness about food safety.	food safety risk transmission perception	China	Analysis of Transmission Model of Consumers' Risk Perception of Food Safety based on Case Analysis	2013
	Incorrect perception of the risks associated with fishery products	fishing products	China	Consumers' perception toward quality and safety of fishery products, Beijing, China	2009
	Risk perceptions can directly affect consumer decision-making behavior	foodborne disease in meat	South Africa	Consumers' Social Representations of Meat Safety in Two Selected Restaurants of Raymond Mhlaba Municipality in the Eastern Cape, South Africa	2017
	Low risk perception about the probability of becoming ill due to the consumption of raw seafood	Seafood	Brazil	Consumption, knowledge, and food safety practices of Brazilian seafood consumers Consumption, knowledge, and food safety practices of Brazilian seafood consumers	2020
	Perceptions of risk in food safety lead to greater willingness to pay. Food safety is the attribute that has the highest premium value accepted by consumers	Pig meat	China	Factoring Chinese consumers' risks perceptions into their willingness to pay for pork safety, environmental stewardship, and animal welfare	2018
	Risk perception has significant effect on	food traceability	China	Factors Affecting Consumer Willingness to Pay for Certified	2021

	willingness to pay a premium on traceable food			Traceable Food in Jiangsu Province of China	
	the more consumers believe that the purchase of chicken products is a risk to some extent during the avian flu period, the more they will reduce their purchase of chicken products.	Purchase intention during the Avian Flu period	China	Avian Influenza-Factors Affecting Consumers' Purchase Intentions toward Poultry Products	2019
	The perception of risk has generally led to decreased demand and increased willingness to pay	Chicken	China	Fear and trust: How risks perceptions of avian influenza affect Chinese consumers' demand for chicken	2016
	Residents felt vulnerable and exposed to threats from unsafe food from the production phase of food supply chains to the food processing, preparation and retail phases.	Perception about food safety condition	China	Food safety in urban China: Perceptions and coping strategies of residents in Nanjing	2017
	Pork and vegetables were considered the least safe food	Perception about food safety condition	China	Food safety in urban China: Perceptions and coping strategies of residents in Nanjing	2017
	Risk perception is impacted by relationship levels (interpersonal, organizational and social);	Traceability system - food safety problem	China		2019
	the social relationship has a significant effect on the risk perception of the group with negative information	Traceability system - food safety problem	China		2019
	Risk perception: for respondents in the negative group, offline social interactions could mitigate the way the public perceives dietary risk, although this influence was weaker than trust, positive emotions and knowledge	Traceability system - food safety problem	China	Are social embeddedness associated with food risk perception under media coverage?	2019
	Risk perception: for respondents in the positive group, interpersonal relationships, social networks and positive emotions can effectively alleviate the risk perception of the results	Traceability system - food safety problem	China		2019
	consumers with different levels of food safety risk perception had dramatically	WTB based on food safety perception	China	Chinese consumer preferences for fresh produce: Interaction between food safety labels and brands	2018

	different perceptions of WTP for the various labels				
	Consumers' willingness to pay for safer food had a slight correlation with perceptions of risk.	foodborne disease	Brazil	Consumer risk perceptions concerning different consequences of Foodborne disease acquired from food consumed away from home: A case study in Brazil	2022
	Participants believe they are less likely than other participants to eat foods that are unsafe	Certified products	China	Optimistic Bias, Food Safety Cognition, and Consumer Behavior of College Students in Taiwan and Mainland China	2020
		Low-cost street food or food services	Brazil	Social representations of safety in food services	2015
	Consumers believe their risk of having a Foodborne disease is lower than other consumers	foodborne disease	Brazil	Knowledge and risk perceptions of Foodborne disease by consumers and food handlers at restaurants with different food safety profiles	2019
	Participants feel in control of their food handling practices, minimizing their personal perception of risk in the home environment.	food contamination	Brazil	Consumer purchase habits and views on food safety: A Brazilian study	2010
**Social pressure**	The greater the social pressure, the greater the search for information on food safety risks	None	China	Food safety risk information-seeking intention of wechat users in China	2020
**Confidence in Food Safety**	The higher the level of confidence in food safety, the lower the risk perception	None	China	Food Safety Trust, Risk Perception, and Consumers' Response to Company Trust Repair Actions in Food Recall Crises	2020
	The higher the level of confidence in food safety, the lower the protective behavioral intention	None	China	Food Safety Trust, Risk Perception, and Consumers' Response to Company Trust Repair Actions in Food Recall Crises	2020
	Greater confidence in guaranteeing security in supermarkets, when compared to open markets.	food contamination	Brazil	Consumer purchase habits and views on food safety: A Brazilian study	2010
	Distrust of packaged and frozen meat cuts found in supermarkets	food contamination	Brazil	Consumer purchase habits and views on food safety: A Brazilian study	2010
	When asked whether information about safety, length of the food chain and traceability would increase their confidence, they responded positively	Chemical and microbiological contamination	Brazil	Consumer perceptions of chemical and microbiological contaminants associated with food chains: A cross-national study	2011
	Confidence in the safety of street foods does not affect the preference for these foods.	Microbiological Hazard	South Africa	The food safety knowledge and microbial hazards awareness of consumers of ready-to-eat street-sold food	2016

	Believing that a brand is less secure can cause consumers to switch brands	food supply chain	China	The factors of consumer confidence recovery after scandals in the food supply chain safety	2018
**Media exposure**	The greater the exposure to the media, the greater the concern with food safety	None	China	Food scandals, media exposure, and citizens' safety concerns: A multilevel analysis across Chinese cities	2016
	Media coverage plays an important role in people's perception of dietary risk after a major food scare	dairy products	China	Understanding Chinese consumers' safety perceptions of dairy products: a qualitative study	2021
	Negative correlation between internet use and gender and urban and rural dwellers (most vulnerable to negative information)	None	China	Association of Internet Use with Attitudes Toward Food Safety in China: A Cross-Sectional Study	2019
	Internet users were more likely to have a lower food safety rating	None	China	Association of Internet Use with Attitudes Toward Food Safety in China: A Cross-Sectional Study	2019
	Internet users are more likely to have a lower food safety rating	None	China	Association of Internet Use with Attitudes Toward Food Safety in China: A Cross-Sectional Study	2019
	Internet use and food safety assessment was significantly different among people of different ages	None	China	Association of Internet Use with Attitudes Toward Food Safety in China: A Cross-Sectional Study	2019
	increased frequency of use of the Internet to search for news, search for information, and browse Weibo lowers people's assessments of food safety	None	China	Association of Internet Use with Attitudes Toward Food Safety in China: A Cross-Sectional Study	2019
	the more doubts people had about the information on the Internet or the opinions of internet users, the greater their assessment of food safety	None	China	Association of Internet Use with Attitudes Toward Food Safety in China: A Cross-Sectional Study	2019
	Internet use had a greater negative impact on people who did not have a rational view of network information	None	China	Association of Internet Use with Attitudes Toward Food Safety in China: A Cross-Sectional Study	2019
	Consumers' perception of risk is influenced by news media, internet and other media	food safety risk transmission perception	China	Analysis of Transmission Model of Consumers' Risk Perception of Food Safety based on Case Analysis	2013
**Concern about food safety**	The greater the concern with risk, the greater the willingness to pay for food safety information	Related to the production process	China	Modeling heterogeneity in consumer preferences for select food safety attributes in China	2011

	Existence of concern with quality and safety issues associated with minimally processed vegetables	food contamination	Brazil	Consumer purchase habits and views on food safety: A Brazilian study	2010
	Greater concern about food hazards of technological origin.	food contamination	Brazil	Consumer purchase habits and views on food safety: A Brazilian study	2010
	Little concern about natural hazards such as microbiological contaminants	food contamination	Brazil	Consumer purchase habits and views on food safety: A Brazilian study	2010
	The need for consumer information on food safety has a significant positive effect on food safety risk communication	Food safety communication on social media	China	Consumers' Food Safety Risk Communication on Social Media Following the Suan Tang Zi Accident: An Extended Protection Motivation Theory Perspective	2021
	Concerns (safety of chicken meat when shopping, during storage and preparation of chicken meat at home, and when consuming chicken meat outside the home) of respondents were inconsistent with their self-reported practices.	Foodborne disease - raw chicken	South Africa	Assessment of safety risks associated with handling chicken as based on practices and knowledge of the group of South African consumers	2019
	Respondents were concerned about sensory evaluation or food quality	Milk and dairy products	India	Customer's Decisiveness About Food Safety Apprehensions of Dairy Products: A Consumer-Centric Approach	2019
	Most respondents were concerned about the dangers of pesticides, followed by illnesses and health disorders	Milk and dairy products	India	Customer's Decisiveness About Food Safety Apprehensions of Dairy Products: A Consumer-Centric Approach	2019
	Respondents are also concerned about the risks related to food packaging, both in terms of sustainability and in terms of food quality	Milk and dairy products	India	Customer's Decisiveness About Food Safety Apprehensions of Dairy Products: A Consumer-Centric Approach	2019
	Chemical waste is the number one food safety concern in the dairy industry	dairy products	China	Exploring Chinese consumers' attitudes toward traceable dairy products: A focus group study	2020
	Beef and pork are, to some extent, associated with health risks, unlike chicken	health risks	Brazil	Beef, chicken and pork consumption and consumer safety and nutritional concerns in the City of Campinas, Brazil	2008
	greater concern with safety attributes than with nutritional attributes	health risks	Brazil	Beef, chicken and pork consumption and consumer safety and nutritional concerns in the City of Campinas, Brazil	2008

	Food safety concerns do not have a major impact on the likelihood of adherence for certification-oriented or price-of-origin-oriented consumers.	Fuji apple	China	Food Safety Concerns and Consumer Preferences for Food Safety Attributes: Evidence from China	2020
	a large proportion of consumers expressed indifference and little or no concern for other relevant food safety issues	Foodborne disease - Food Truck	Brazil	Brazilian Food Truck Consumers' Profile, Choices, Preferences, and Food Safety Importance Perception	2019
	The perception of the importance of food safety for food safety training did not differ between education level, gender or marital status;	Foodborne disease - Food Truck	Brazil	Brazilian Food Truck Consumers' Profile, Choices, Preferences, and Food Safety Importance Perception	2019
	Most were concerned about the use of gloves, masks, caps, the existence of a sink for hand washing, correct use of garbage collectors and the absence of pests and vectors in the preparation area	Foodborne disease - Food Truck	Brazil	Brazilian Food Truck Consumers' Profile, Choices, Preferences, and Food Safety Importance Perception	2019
	Concern for fresh food sold locally increases with awareness of the hygiene of places where fresh food is sold	Foodborne disease - awareness	China	Chinese consumers food purchasing behaviors and awareness of food safety	2017
	Consumers say that meat and contamination during transport pose a greater risk to safety; less concern with vegetables and fruits; concerned about contamination during the transport of frozen food and suspicions about shelf life and storage conditions	foodborne disease	China	Chinese consumers' perceptions of food safety cues and maximizing the effectiveness of food safety communications	2020
	Additives and GM major associations with food safety; fresh food has less additives; associate "sanitation" with the hygiene condition of food; food safety is related to health and nutrition.	foodborne disease	China	Chinese consumers' perceptions of food safety cues and maximizing the effectiveness of food safety communications	2020
	Both groups (high and low anchoring) expressed concern about FBD before the intervention and it	foodborne disease	China	Cognitive Biases of Consumers' Risk Perception of Foodborne Diseases in China: Examining Anchoring Effect	2019

	increased with the intervention.				
	Perception of sanitation at the place of purchase: supermarket group - I was more satisfied	meat safety	Brazil	Concerns, attitudes, and opinions of meat buyers in Garanhuns, Pernambuco, Brazil	2021
	Recall was perceived as a sign of recognition by the producer of consumer concerns	Chemical and microbiological contamination	Brazil	Consumer perceptions of chemical and microbiological contaminants associated with food chains: A cross-national study	2011
	powdered milk can be easily contaminated by chemicals used in the formation phase process; the chicken chain is more vulnerable; farmed salmon: chemical and microbiological contaminants were considered risky; Drinking water: considered that the drinking water chain is subject to contamination from a variety of contaminants	Chemical and microbiological contamination	Brazil	Consumer perceptions of chemical and microbiological contaminants associated with food chains: A cross-national study	2011
	greater concern with different types of additives and more chemical than microbiological contamination	Chemical and microbiological contamination	Brazil	Consumer perceptions of chemical and microbiological contaminants associated with food chains: A cross-national study	2011
	considered all meat products were risky in terms of contamination, fruits and vegetables were considered less risky and dry food products were considered less vulnerable to contamination;	Chemical and microbiological contamination	Brazil	Consumer perceptions of chemical and microbiological contaminants associated with food chains: A cross-national study	2011
	food products are exposed to potential contamination risks	Chemical and microbiological contamination	Brazil	Consumer perceptions of chemical and microbiological contaminants associated with food chains: A cross-national study	2011
	Most Chinese consumers are concerned about food safety issues	food traceability	China	Factors Affecting Consumer Willingness to Pay for Certified Traceable Food in Jiangsu Province of China	2021
	Degree of concern has significant effect on willingness to pay a premium on traceable food	food traceability	China	Factors Affecting Consumer Willingness to Pay for Certified Traceable Food in Jiangsu Province of China	2021

	If consumers have a higher level of concern about the safety of vegetables, there is a higher purchase intention and willingness to pay a premium price for safe vegetables.	Vegetables	China	Consumers' perceptions, purchase intention, and willingness to pay a premium price for safe vegetables: A case study of Beijing, China	2018
	most were concerned about food safety (attitude towards purchase)	meat safety	Brazil	Concerns, attitudes, and opinions of meat buyers in Garanhuns, Pernambuco, Brazil	2021
	food safety had the greatest influence on the general attitude towards different pig production systems.	swine	China	Consumer attitudes to different pig production systems: a study from mainland China	2013
	Brazilians indicated that they are more concerned about food safety and hygiene practices in the context of COVID-19	COVID-19	Brazil	Effect of the COVID-19 pandemic on food habits and perceptions: A study with Brazilians	2021
**Frequency of consumption**	The higher the frequency of consumption, the greater the concern with milk safety	Milk	China	Perceived risks and safety concerns about fluid milk among Chinese college students	2010
	The greater the expenditure on food in the family, the greater the intention to buy safe vegetables	Vegetables	China	Consumers' perceptions, purchase intention, and willingness to pay a premium price for safe vegetables: A case study of Beijing, China	2018
**Subjective norm**	The higher the subjective norm, the greater the perception of risk	Additions	China	Public risk perception of food additives and food scares. The case in Suzhou, China	2013
	Subjective norm is a significant predictor of intention to hygienic food handling	Intention of hygienic handling of food	China	Hygienic food handling intention. An application of the Theory of Planned Behavior in the Chinese cultural context	2014
	Influence of media reports on consumers is positively associated with purchase intentions	Purchase intention during the Avian Flu period	China	Avian Influenza-Factors Affecting Consumers' Purchase Intentions toward Poultry Products	2019
**Perception of information**	The greater the perception of information, the greater the perception of risk	Additions	China	Public risk perception of food additives and food scares. The case in Suzhou, China	2013
	Perception of information: the origin of chicken products is essential for consumers	Purchase intention during the Avian Flu period	China	Avian Influenza-Factors Affecting Consumers' Purchase Intentions toward Poultry Products	2019
	Most respondents believe that the information available is not sufficient to judge the safety of a food.	Milk and dairy products	India	Customer's Decisiveness About Food Safety Apprehensions of Dairy Products: A Consumer-Centric Approach	2019
	Preference for information sources that are empirically proven to significantly	Risk information sources	China	Does Information Pattern Affect Risk Perception of Food Safety? The National Survey in China	2018

	affect your perceptions of food safety				
	Social media is the main source of misinformation about food safety	Dissemination of disinformation	China	Fake news or bad news? Toward an emotion-driven cognitive dissonance model of misinformation diffusion	2020
	Exposure to online news reduces levels of misinformation	Dissemination of disinformation	China	Fake news or bad news? Toward an emotion-driven cognitive dissonance model of misinformation diffusion	2020
	Trust in information sources is a strong determinant of consumer response in terms of consumption intention or willingness to pay	Chicken	China	Fear and trust: How risks perceptions of avian influenza affect Chinese consumers' demand for chicken	2016
**Experience (direct or indirect) with a food safety incident**	Consumers who have had direct experiences with food safety incidents know more about food safety and are more familiar with it.	None	China	Research on the Influence Mechanism of Rational Consumers' Food Safety Supervision Satisfaction	2019
	Defensive psychological state after severe food quality safety crises	None	China	Why Do Consumers Switch in Food Safety Crisis? A View of Regulatory Focus Theory	2015
	Food safety incidents can significantly affect consumers' purchasing response, increasing the purchase of organic products	Organic products	China	The Predictors of Consumer Behavior in Relation to Organic Food in the Context of Food Safety Incidents: Advancing Hyper Attention Theory Within an Stimulus-Organism-Response Model	2019
	Previous negative experience with Foodborne disease increases the perception of risk of Foodborne disease	foodborne disease	China	Consumer's risk perception of Foodborne diseases and high-risk food safety practices in domestic kitchens	2019
	The perceived severity of a previous food safety accident has a positive effect on consumer protection motivation	Food safety communication on social media	China	Consumers' Food Safety Risk Communication on Social Media Following the Suan Tang Zi Accident: An Extended Protection Motivation Theory Perspective	2021
	Few consumers know details of incidents about the quality and safety of fish products	fishing products	China	Consumers' perception toward quality and safety of fishery products, Beijing, China	2009
	The greater the understanding of past background related to meat food safety, the richer the knowledge about food safety and the more attentive to food risks	foodborne disease in meat	South Africa	Consumers' Social Representations of Meat Safety in Two Selected Restaurants of Raymond Mhlaba Municipality in the Eastern Cape, South Africa	2017
	The prevalence of food safety incidents causes consumers to lessen their	dairy products	China	Exploring Chinese consumers' attitudes toward traceable dairy products: A focus group study	2020

	confidence in food safety and pay more attention to news about food safety incidents in the media, including social media.				
	Chicken consumption did not increase after the appearance of H7N9	Chicken	China	Fear and trust: How risks perceptions of avian influenza affect Chinese consumers' demand for chicken	2016
	Food safety incident triggers the risk of price changes for high-risk food products but not low-risk food products	Reactions to food safety incidents	China	Food Safety Incident, Public Health Concern, and Risk Spillover Heterogeneity: Avian Influenza Shocks as Natural Experiments in China's Consumer Markets	2019
	Food safety incidents trigger, amplify public health concerns and affect food prices	Reactions to food safety incidents	China	Food Safety Incident, Public Health Concern, and Risk Spillover Heterogeneity: Avian Influenza Shocks as Natural Experiments in China's Consumer Markets	2019
	Dairy security incidents (especially melamine incidents) increase urban residents' perception of risk, resulting in a rapid decrease in dairy consumption.	dairy	China	Food safety issues in China: a case study of the dairy sector	2015
	The impact of the dairy safety incidents lasted for at least 5 years and the consumption of dairy products has not fully recovered by the date of publication of the article.	dairy	China	Food safety issues in China: a case study of the dairy sector	2015
	food marketing declined if anyone had heard of reports of food safety incidents	Foodborne disease - awareness	China	Chinese consumers food purchasing behaviors and awareness of food safety	2017
**Trust in Government and Policies**	The greater the confidence in the government, the lower the perception of risk on food safety	None	China	Research on the influencing factors of consumer food safety risk perception	2017
	Government regulation on food safety and consumer protection is an attribute of low importance for food purchasing decision making.	regulation	India	Consumers' Perspectives on Mandatory versus Voluntary Provisions of Food Safety Regulations in India	2013
	Consumers believe government must play a critical role in providing safe food	regulation	India	Consumers' Perspectives on Mandatory versus Voluntary Provisions of Food Safety Regulations in India	2013
	Consumers who prefer to assess product quality prior to purchase (search	regulation	India	Consumers' Perspectives on Mandatory versus Voluntary Provisions of Food Safety Regulations in India	2013

	attribute) believe that voluntary provisions are sufficient for effective regulation of food safety. Consumers who prefer certifications and regulatory practices (credence attribute) understand that the combination of voluntary and mandatory provisions need to be implemented for an effective regulation of food safety [in this case, mandatory regulation is of paramount importance].				
	Low satisfaction from government oversight and food producers' control over food quality, yet consumers are concerned about meat food safety issues	foodborne disease in meat	South Africa	Consumers' Social Representations of Meat Safety in Two Selected Restaurants of Raymond Mhlaba Municipality in the Eastern Cape, South Africa	2017
	The distrust in the government will increase the consumption intention and the willingness to pay	Chicken	China	Fear and trust: How risks perceptions of avian influenza affect Chinese consumers' demand for chicken	2016
	Consensus of participants that local government is ineffective in regulation, causing food safety problems	Perception about food safety condition	China	Food safety in urban China: Perceptions and coping strategies of residents in Nanjing	2017
	When consumers realized that food safety authorities had taken steps to proactively protect the public, they rated FRMQ (Quality in Food Risk Management) more positively;	None	Russia	Consumer evaluations of food risk management in Russia	2010
	Russian consumers were not confident that food safety related laws and regulations were being enforced	None	Russia	Consumer evaluations of food risk management in Russia	2010
	perception that the competent authorities could not or were not willing to take responsibility for all problems related to food safety	None	Russia	Consumer evaluations of food risk management in Russia	2010
**Confidence in the Media**	The greater the confidence in the media, the lower the	None	China	Research on the influencing factors of consumer food safety risk perception	2017

	perception of food safety risk				
	Awareness of the value of news, trust and emotion reduces respondents' perception of probability risk	Traceability system - food safety problem	China	Are social embeddedness associated with food risk perception under media coverage?	2019
**Trust in manufacturers and retailers**	The greater the trust in manufacturers and retailers, the greater the perception of food safety	None	China	The effects of different types of trust on consumer perceptions of food safety: An empirical study of consumers in Beijing Municipality, China	2013
	The brand is of great importance in the purchase decision process	dairy products	China	Understanding Chinese consumers' safety perceptions of dairy products: a qualitative study	2021
	Familiarity with the butcher is seen as a way to build consumer confidence.	food contamination	Brazil	Consumer purchase habits and views on food safety: A Brazilian study	2010
	Perceptions of personal hygiene at the place of meat purchase plays an important role in ensuring safe products for the consumer	foodborne disease in meat	South Africa	Consumers' Social Representations of Meat Safety in Two Selected Restaurants of Raymond Mhlaba Municipality in the Eastern Cape, South Africa	2017
	The production and marketing of vegetables is the method used to assess safe vegetables	Vegetables	China	Consumers' perceptions, purchase intention, and willingness to pay a premium price for safe vegetables: A case study of Beijing, China	2018
	high level of experience perceived by consumers as possessed by food risk managers was associated with a more positive assessment of the FRMQ (quality in food risk management)	None	Russia	Consumer evaluations of food risk management in Russia	2010
	the more consumers perceived risk managers as dishonest, the less they believed the FRMQ was adequate.	None	Russia	Consumer evaluations of food risk management in Russia	2010
	the large-scale industrial food production system is generally well accepted as it appears to be a pathway to food safety,	swine	China	Consumer attitudes to different pig production systems: a study from mainland China	2013
	Chinese were more positive about pig production systems where "food safety receives the utmost attention when raising pigs on this farm	swine	China	Consumer attitudes to different pig production systems: a study from mainland China	2013

Confidence in certification / perception of certification	Consumers demand safe food but do not fully trust food certifications as a guarantee of safety	certified food	China	Urban Consumer Trust and Food Certifications in China	2020
	The expiration dates shown on the meat packages were more important measures in meat spoilage than the visual appearance or color of the meat.	foodborne disease in meat	South Africa	Consumers' Social Representations of Meat Safety in Two Selected Restaurants of Raymond Mhlaba Municipality in the Eastern Cape, South Africa	2017
	Most consumers use certification labels on vegetable packaging to identify safe vegetables	Vegetables	China	Consumers' perceptions, purchase intention, and willingness to pay a premium price for safe vegetables: A case study of Beijing, China	2018
	Confidence in the label had a significant positive effect on purchase intent and willingness to pay a premium price for safe vegetables	Vegetables	China	Consumers' perceptions, purchase intention, and willingness to pay a premium price for safe vegetables: A case study of Beijing, China	2018
	Respondents have doubts about the veracity of traceability information	dairy products	China	Exploring Chinese consumers' attitudes toward traceable dairy products: A focus group study	2020
	Foods with national and North American labels have greater acceptance for premium price among Chinese consumers	Pig meat	China	Factoring Chinese consumers' risks perceptions into their willingness to pay for pork safety, environmental stewardship, and animal welfare	2018
	Chinese Consumers Value Government Certification More	Fuji apple	China	Food Safety Concerns and Consumer Preferences for Food Safety Attributes: Evidence from China	2020
	Chinese consumers may not understand the meaning of safety rating notifications and official inspection certifications and therefore rarely use them to assess the safety of restaurant food	Restaurants	China	Food safety in restaurants: The consumer perspective	2019
	The participants' perception is that food monitoring and regulation is not adequate to ensure the safety of food sources	Perception about food safety condition	China	Food safety in urban China: Perceptions and coping strategies of residents in Nanjing	2017
	Certification-oriented consumers have a high assessment of food safety and government oversight of food safety. Price- and origin-oriented consumers only place a high value on	Fuji apple	China	Food Safety Concerns and Consumer Preferences for Food Safety Attributes: Evidence from China	2020

	the local assessment of food security.				
Loss aversion	Loss aversion influences consumers in their food choices for food safety	Food safety (certification and traceability) vs. Quality labels (brand and category)	China	Influence of Loss Aversion and Income Effect on Consumer Food Choice for Food Safety and Quality Labels	2021
	Concerning the wage discount if there is a Foodborne disease, the perception of risk is not greater because the consumer has to stop working for one to three days, but because of the financial consequences	foodborne disease	Brazil	Consumer risk perceptions concerning different consequences of Foodborne disease acquired from food consumed away from home: A case study in Brazil	2022
	Financial loss resulting from medications or medical treatments has less impact than the physical consequences of a Foodborne disease	foodborne disease	Brazil	Consumer risk perceptions concerning different consequences of Foodborne disease acquired from food consumed away from home: A case study in Brazil	2022
	Perceived vulnerability in relation to food safety has a significant positive effect on consumer protection motivation	Food safety communication on social media	China	Consumers' Food Safety Risk Communication on Social Media Following the Suan Tang Zi Accident: An Extended Protection Motivation Theory Perspective	2021
	The probability of illness impacts the preference for obtaining safe food products	Pig meat	China	Factoring Chinese consumers' risks perceptions into their willingness to pay for pork safety, environmental stewardship, and animal welfare	2018
Safety Perception	Consumers are willing to pay a premium price for a safer meal	foodborne disease	Brazil	Consumer risk perceptions concerning different consequences of Foodborne disease acquired from food consumed away from home: A case study in Brazil	2022
	The willingness to pay for safe, high-quality fruit decreases as prices rise, so consumers are willing to pay a premium for "safe grapes", but within a scope	grape products	China	Consumers' perceptions of quality and safety for grape products: A case study in Zhejiang Province, China	2012
	Most consumers are willing to buy safe vegetables, including paying a premium price.	Vegetables	China	Consumers' perceptions, purchase intention, and willingness to pay a premium price for safe vegetables: A case study of Beijing, China	2018
	Willingness to pay for safe chicken has not increased compared to generic risks associated with chicken consumption in 2012	Chicken	China	Fear and trust: How risks perceptions of avian influenza affect Chinese consumers' demand for chicken	2016

	Willingness to pay a premium price for apples' safety attributes	Fuji apple	China	Food Safety Concerns and Consumer Preferences for Food Safety Attributes: Evidence from China	2020
	The majority of those who have consumed insects believe that this source is safe, very safe or extremely safe and a minority claimed to be unsafe, very unsafe or extremely unsafe.	Insect consumption safety	Brazil	Brazilian consumers' perception of edible insects	2019
	Most respondents do not consider domestically produced cheese as a risky product in terms of food safety, but of a lower quality than imported cheese.	national x imported cheese	Russia	Food quality vs food patriotism: Russian consumers' preferences for cheese after the food import ban	2018
	The three main advantages perceived by consumers with respect to safe vegetables are: food safety, rigorous production and processing, and a healthy environment.	Vegetables	China	Consumers' perceptions, purchase intention, and willingness to pay a premium price for safe vegetables: A case study of Beijing, China	2018
	Greater perception of quality and safety with respect to imported grapes	grape products	China	Consumers' perceptions of quality and safety for grape products: A case study in Zhejiang Province, China	2012
	The perception of safety is important in evaluating the quality of grape products.	grape products	China	Consumers' perceptions of quality and safety for grape products: A case study in Zhejiang Province, China	2012
	The three main advantages perceived by consumers with respect to safe vegetables are: food safety, rigorous production and processing, and a healthy environment.	Vegetables	China	Consumers' perceptions, purchase intention, and willingness to pay a premium price for safe vegetables: A case study of Beijing, China	2018
	The perception of the safety status of vegetables has a significant negative effect on purchase intent and willingness to pay a premium price for safe vegetables.	Vegetables	China	Consumers' perceptions, purchase intention, and willingness to pay a premium price for safe vegetables: A case study of Beijing, China	2018
	Purchase awareness (freshness, warranty period and expiration date, date of harvest, nutritional health, production area, packaging, trusted label, media promotion, recommendation from	Vegetables	China	Consumers' perceptions, purchase intention, and willingness to pay a premium price for safe vegetables: A case study of Beijing, China	2018

	others, online shopping experience) influenced purchase intent and disposition of paying a premium price for safe vegetables				
	The more consumers believe that the purchase of chicken products can pose a threat to their health, the more they will reduce their purchase of chicken products.	Purchase intention during the Avian Flu period	China	Avian Influenza-Factors Affecting Consumers' Purchase Intentions toward Poultry Products	2019
	consumers believe that during the bird flu period they will, to some extent, reduce their purchases of chicken products.	Purchase intention during the Avian Flu period	China	Avian Influenza-Factors Affecting Consumers' Purchase Intentions toward Poultry Products	2019
**Knowledge**	Knowledge of traceable dairy products is low	dairy products	China	Exploring Chinese consumers' attitudes toward traceable dairy products: A focus group study	2020
	Greater knowledge about pathogens or professional knowledge about food safety increases risk perception of Foodborne disease	foodborne disease	China	Consumer's risk perception of Foodborne diseases and high-risk food safety practices in domestic kitchens	2019
	Low knowledge about quality, safety and traceability systems of fishery products	fishing products	China	Consumers' perception toward quality and safety of fishery products, Beijing, China	2009
	Consumers with greater knowledge about safe vegetables are more likely to have a higher purchase intention and willingness to pay a premium price for safe vegetables	Vegetables	China	Consumers' perceptions, purchase intention, and willingness to pay a premium price for safe vegetables: A case study of Beijing, China	2018
	Most consumers are unaware of the safety and quality of meat	Beef	India	Consumption pattern, consumer attitude and consumer perception on meat quality and safety in Southern India	2018
	The vast majority of respondents do not attribute meat consumption to any type of food poisoning	Beef	India	Consumption pattern, consumer attitude and consumer perception on meat quality and safety in Southern India	2018
	Most respondents reported not having heard about Vibrio and Listeria monocytogenes as bacterial	Seafood	Brazil	Consumption, knowledge, and food safety practices of Brazilian seafood consumers Consumption, knowledge, and food safety practices of Brazilian seafood consumers	2020

	pathogens associated with seafood consumption				
	Lack of knowledge and skills in food handling behavior is the main reason for not adopting proper food safety self-protection behavior.	foodborne disease	China	Determinants of consumer food safety self-protection behavior-- an analysis using grounded theory	2020
	Knowledge of food safety affects self-protection behavior through self-protection awareness	foodborne disease	China	Determinants of consumer food safety self-protection behavior-- an analysis using grounded theory	2020
	Individuals who have more erroneous information disclose (mis)information about food safety more often online	Dissemination of disinformation	China	Fake news or bad news? Toward an emotion-driven cognitive dissonance model of misinformation diffusion	2020
	The majority has no opinion about safety and the minority believes it is safe.	Insect consumption safety	Brazil	Brazilian consumers' perception of edible insects	2019
	Among the participants who do not consume insects, most do not have an opinion and of those who do, most claim to be unsafe, very unsafe or extremely unsafe	Insect consumption safety	Brazil	Brazilian consumers' perception of edible insects	2019
	Knowledge about food safety and the choice of food purchased are related to sociodemographic factors	Foodborne disease - awareness	China	Chinese consumers food purchasing behaviors and awareness of food safety	2017
	Most are familiar with the country of origin, traceability, organic and quality attribute terminology certifications from the dairy farm itself;	powdered milk safety	China	Chinese consumers' preferences for food quality test/measurement indicators and cues of milk powder: A case of Zhengzhou, China	2019
	FBD knowledge cannot effectively reduce the external anchoring effect	foodborne disease	China	Cognitive Biases of Consumers' Risk Perception of Foodborne Diseases in China: Examining Anchoring Effect	2019
	The majority reported consuming inspected meat or having undergone a quality control process (Knowledge about inspection/quality control process)	meat safety	Brazil	Concerns, attitudes, and opinions of meat buyers in Garanhuns, Pernambuco, Brazil	2021
	Most claimed to know the risks and had the understanding that the consumption of	meat safety	Brazil	Concerns, attitudes, and opinions of meat buyers in Garanhuns, Pernambuco, Brazil	2021

	unregulated meat could result in the transmission of diseases				
	More than half knew that pork with cysticercosis could be harmful to health and could identify cysts when slaughtering pigs at home, but were unaware of the symptoms related to human cysticercosis;	Biological contamination (Taenia solium)	South Africa	Consumer knowledge and practices for pork safety in two Taenia solium cysticercosis endemic districts in Eastern Cape Province of South Africa	2020
	consumers were unaware of existing meat/food safety legislations	Biological contamination (Taenia solium)	South Africa	Consumer knowledge and practices for pork safety in two Taenia solium cysticercosis endemic districts in Eastern Cape Province of South Africa	2020
**Eating habits and health**	The frequency of cooking, cleaning of cutting tools and cutting boards and the storage time of food at room temperature were independent of the risk perception of Foodborne disease	foodborne disease	China	Consumer's risk perception of Foodborne diseases and high-risk food safety practices in domestic kitchens	2019
	Consumers with good hygiene and eating habits have a higher perception of risk for Foodborne disease	foodborne disease	China	Consumer's risk perception of Foodborne diseases and high-risk food safety practices in domestic kitchens	2019
	Most consumers return the product, followed by discarding the product and complaining to the competent body.	fishing products	China	Consumers' perception toward quality and safety of fishery products, Beijing, China	2009
	Most interviewees stated that they adopted some hygienic practice when handling seafood (washing hands with soap; the majority claimed to separate raw foods from ready-to-eat foods)	Seafood	Brazil	Consumption, knowledge, and food safety practices of Brazilian seafood consumers Consumption, knowledge, and food safety practices of Brazilian seafood consumers	2020
	The reliability of consumers' attitude towards food safety practices is high	Milk and dairy products	India	Customer's Decisiveness About Food Safety Apprehensions of Dairy Products: A Consumer-Centric Approach	2019
	Personality (including personal handling habits, laziness, and past experiences) directly affects self-protective behavior and moderates the effects of knowledge and awareness on behavior.	foodborne disease	China	Determinants of consumer food safety self-protection behavior-- an analysis using grounded theory	2020

	The thawing of chicken meat, the time and temperature of egg cooking and the yolk point are the main procedures responsible for raising the level of WHOPI (Weighted Harmonic Outbreak Prevention Index)	chickens and eggs	Brazil	Food safety behavior and handling practices during purchase, preparation, storage and consumption of chicken meat and eggs	2019
	Most respondents had an average level of good hygiene practices procedures	chickens and eggs	Brazil	Food safety behavior and handling practices during purchase, preparation, storage and consumption of chicken meat and eggs	2019
	Chinese consumers prefer to assess the level of safety of a restaurant's food based on the "sensory perception of the food" and the "restaurant environment"	Restaurants	China	Food safety in restaurants: The consumer perspective	2019
	The way to understand whether a food is safe is by looking for signs that the food is fresh and by looking for information on which synthetic products were or were not used in the production of that food.	Perception about food safety condition	China	Food safety in urban China: Perceptions and coping strategies of residents in Nanjing	2017
	Most preferred the pork to be well cooked (well done), they indicated that the main reason for cooking “well done” is that it killed the germs.	Biological contamination (Taenia solium)	South Africa	Consumer knowledge and practices for pork safety in two Taenia solium cysticercosis endemic districts in Eastern Cape Province of South Africa	2020
**Probabilities and consequences**	Probability and consequence measures of Foodborne disease modulate risk perception	foodborne disease	Brazil	Consumer risk perceptions concerning different consequences of Foodborne disease acquired from food consumed away from home: A case study in Brazil	2022
	Consequences related to performance, physical and time increase the perception of risk	foodborne disease	Brazil	Consumer risk perceptions concerning different consequences of Foodborne disease acquired from food consumed away from home: A case study in Brazil	2022
**Self-efficacy and response effectiveness**	Self-efficacy (in relation to food safety) has a significant positive effect on consumer protection motivation	Food safety communication on social media	China	Consumers' Food Safety Risk Communication on Social Media Following the Suan Tang Zi Accident: An Extended Protection Motivation Theory Perspective	2021
	Response effectiveness in relation to food safety) has a positive influence on consumer protection motivation	Food safety communication on social media	China	Consumers' Food Safety Risk Communication on Social Media Following the Suan Tang Zi Accident: An Extended Protection Motivation Theory Perspective	2021

	The self-efficacy of the Internet reduces levels of misinformation, but it also facilitates the dissemination of information on food safety	Dissemination of disinformation	China	Fake news or bad news? Toward an emotion-driven cognitive dissonance model of misinformation diffusion	2020
**Response barrier**	Response barriers (perceived costs associated with food safety protection measures or actions) have a negative influence on consumer protection motivation	Food safety communication on social media	China	Consumers' Food Safety Risk Communication on Social Media Following the Suan Tang Zi Accident: An Extended Protection Motivation Theory Perspective	2021
**Protection motivation**	Consumer protection motivation has a significant positive influence on food safety risk communication on social media	Food safety communication on social media	China	Consumers' Food Safety Risk Communication on Social Media Following the Suan Tang Zi Accident: An Extended Protection Motivation Theory Perspective	2021
**Emotions**	Negative emotions mediate levels of misinformation and dissemination of food safety information	Dissemination of disinformation	China	Fake news or bad news? Toward an emotion-driven cognitive dissonance model of misinformation diffusion	2020
	Fear of the spread of H7N9 increases consumption intent and willingness to pay	Chicken	China	Fear and trust: How risks perceptions of avian influenza affect Chinese consumers' demand for chicken	2016

Table S4 – Motivators and effects of "other" category

Motivator	Observed effect	Hazard	Country	Study title	Year
**Price**	A greater fluctuation in the price of vegetables results in a lesser willingness to pay a premium price for safe vegetables	Vegetables	China	Consumers' perceptions, purchase intention, and willingness to pay a premium price for safe vegetables: A case study of Beijing, China	2018
	Consumers who see higher vegetable prices are no longer likely to buy and are not willing to pay a premium price for safe vegetables	Vegetables	China	Consumers' perceptions, purchase intention, and willingness to pay a premium price for safe vegetables: A case study of Beijing, China	2018
	After the price reaches a peak level that indicates a certain quality standard, the price-value effect will dominate the price-quality effect, thus decreasing the utility for the consumer;	powdered milk safety	China	Chinese consumers' preferences for food quality test/measurement indicators and cues of milk powder: A case of Zhengzhou, China	2019
	If the price is too low (45 RMB), the consumer considers the powdered milk to be of low quality; if the price peaks (122.2 RMB), the price-quality signaling effect decreases and the price starts to have a negative impact on the probability of purchasing powdered milk	powdered milk safety	China	Chinese consumers' preferences for food quality test/measurement indicators and cues of milk powder: A case of Zhengzhou, China	2019
	Price- and origin-oriented consumers place a high value on the local food safety assessment.	Fuji apple	China	Food Safety Concerns and Consumer Preferences for Food Safety Attributes: Evidence from China	2020
**Traceability**	Most respondents are willing to pay a premium for the fish products traceability program to increase safety. The average premium price for respondents was 6%.	fishing products	China	Consumers' perception toward quality and safety of fishery products, Beijing, China	2009
	The willingness to pay a premium amount for certified traceable food is limited.	food traceability	China	Factors Affecting Consumer Willingness to Pay for Certified Traceable Food in Jiangsu Province of China	2021
	The perception of quality and safety is not a consequence of the traceability of grape foods in the consumers' perception	grape products	China	Consumers' perceptions of quality and safety for grape products: A case study in Zhejiang Province, China	2012
**Eating habits and health**	The frequency of cooking, cleaning of cutting tools and cutting boards, and the storage time of food at room	foodborne disease	China	Consumer's risk perception of Foodborne diseases and high-risk	2019

	temperature was independent of the risk perception of Foodborne disease			food safety practices in domestic kitchens	
	Consumers with good hygiene and eating habits have a higher perception of risk for Foodborne disease	foodborne disease	China	Consumer's risk perception of Foodborne diseases and high-risk food safety practices in domestic kitchens	2019
	Most consumers return or discard the product and complain to the competent body.	fishing products	China	Consumers' perception toward quality and safety of fishery products, Beijing, China	2009
	Most interviewees stated that they adopted some hygienic practice when handling seafood (washing hands with soap; the majority claimed to separate raw foods from ready-to-eat foods)	Seafood	Brazil	Consumption, knowledge, and food safety practices of Brazilian seafood consumers Consumption, knowledge, and food safety practices of Brazilian seafood consumers	2020
	The reliability of consumers' attitudes towards food safety practices is high	Milk and dairy products	India	Customer's Decisiveness About Food Safety Apprehensions of Dairy Products: A Consumer-Centric Approach	2019
	Personality (including personal handling habits, laziness, and past experiences) directly affects self-protective behavior and moderates the effects of knowledge and awareness on behavior.	foodborne disease	China	Determinants of consumer food safety self-protection behavior-- an analysis using grounded theory	2020
	The thawing of chicken meat, the time and temperature of egg cooking and the yolk point are the main procedures responsible for raising the level of WHOPI (Weighted Harmonic Outbreak Prevention Index)	chickens and eggs	Brazil	Food safety behavior and handling practices during purchase, preparation, storage and consumption of chicken meat and eggs	2019
	Most respondents had an average level of good hygiene practices procedures	chickens and eggs	Brazil	Food safety behavior and handling practices during purchase, preparation, storage and consumption of chicken meat and eggs	2019
	Chinese consumers prefer to assess the level of safety of a restaurant's food based on the "sensory perception of the food" and the "restaurant environment"	Restaurants	China	Food safety in restaurants: The consumer perspective	2019
	The way to understand whether a food is safe is by looking for signs that the food is fresh and by looking for	Perception about food	China	Food safety in urban China: Perceptions and coping strategies of residents in Nanjing	2017

	information on which synthetic products were or were not used to produce that food.	safety condition			
	Most preferred the pork to be well cooked (well done). They indicated that the main reason for cooking "well done" is that it killed the germs.	Biological contamination (Taenia solium)	South Africa	Consumer knowledge and practices for pork safety in two Taenia solium cysticercosis endemic districts in Eastern Cape Province of South Africa	2020
**Place of consumption / purchase / physical environment**	For the consumer, the probability of having a Foodborne disease is greater in another restaurant than in the current restaurant.	foodborne disease	Brazil	Consumer risk perceptions concerning different consequences of Foodborne disease acquired from food consumed away from home: A case study in Brazil	2022
	Consumers underestimate the risk of foodborne disease when eating out	foodborne disease	Brazil	Consumer risk perceptions concerning different consequences of Foodborne disease acquired from food consumed away from home: A case study in Brazil	2022
	Consumers realize that the risks of dining at the current establishment are similar to those posed by dining in their own homes	foodborne disease	Brazil	Consumer risk perceptions concerning different consequences of Foodborne disease acquired from food consumed away from home: A case study in Brazil	2022
	Different types of restaurants lead to a shift in consumers' perception of risk	foodborne disease	Brazil	Consumer risk perceptions concerning different consequences of Foodborne disease acquired from food consumed away from home: A case study in Brazil	2022
	Consumers prefer to buy meat at upper-class retailers and believe that this point sells safer meats	foodborne disease in meat	South Africa	Consumers' Social Representations of Meat Safety in Two Selected Restaurants of Raymond Mhlaba Municipality in the Eastern Cape, South Africa	2017
	The lack of structure in the environments (presence of a refrigerator, running water...) restricts self-protection behaviors	foodborne disease	China	Determinants of consumer food safety self-protection behavior-- an analysis using grounded theory	2020
	Most have the perception that street foods are partially clean	foodborne disease - street food	Brazil	Characterization of street food consumption according to age, multicentric study	2018
	The hygiene of the place of sale and the freshness of the meat were the most	foodborne disease in meat	South Africa	Consumers' Social Representations of Meat Safety in Two Selected Restaurants of Raymond Mhlaba	2017

	important factors for consumers in relation to the meat safety assessment		Municipality in the Eastern Cape, South Africa
